# Extracellular Vesicles Carrying Tenascin-C are Clinical Biomarkers and Improve Tumor-Derived DNA Analysis in Glioblastoma Patients

**DOI:** 10.1021/acsnano.4c13599

**Published:** 2025-03-08

**Authors:** Amanda Salviano-Silva, Kathrin Wollmann, Santra Brenna, Rudolph Reimer, Julia E. Neumann, Matthias Dottermusch, Laura Woythe, Cecile L. Maire, Berta Puig, Ulrich Schüller, Meike J. Saul, Manfred Westphal, Richard Drexler, Lasse Dührsen, Jens Gempt, Dieter H. Heiland, Katrin Lamszus, Franz L. Ricklefs

**Affiliations:** † Department of Neurosurgery, University Medical Center Hamburg-Eppendorf, Hamburg 20246, Germany; ‡ Neurology Department, Experimental Research in Stroke and Inflammation, University Medical Center Hamburg-Eppendorf, Hamburg 20246, Germany; § Leibniz Institute for Experimental Virology, Hamburg 20251, Germany; ∥ Institute of Neuropathology, University Medical Center Hamburg-Eppendorf, Hamburg 20246, Germany; ⊥ Center for Molecular Neurobiology (ZMNH), University Medical Center Hamburg-Eppendorf, Hamburg 20246, Germany; # Oxford Nanoimaging Limited (ONI), Oxford OX2 8TA, U.K.; ¶ Department of Pediatric Hematology and Oncology, University Medical Center Hamburg-Eppendorf, 20246 Hamburg, Germany; ∇ Children’s Cancer Research Center Hamburg, Hamburg 20246, Germany; ○ Department of Oncology, Hematology and Bone Marrow Transplantation with Section Pneumology, University Cancer Center Hamburg, University Clinic Hamburg-Eppendorf, Hamburg 20246, Germany; ⧫ Department of Neurosurgery, Medical Center University of Freiburg, Freiburg D-79106, Germany; †† Translational Neurosurgery, Friedrich-Alexander University Erlangen Nuremberg, Erlangen 91054, Germany; ‡‡ Department of Neurosurgery, University Hospital Erlangen, Friedrich-Alexander University Erlangen Nuremberg, Erlangen 91054, Germany; §§ Department of Neurological Surgery, Northwestern University Feinberg School of Medicine, Chicago, Illinois 60611, United States; ∥∥ German Cancer Consortium (DKTK), Partner Site Freiburg, Freiburg D-79106, Germany

**Keywords:** Tenascin-C, extracellular vesicles, glioblastoma, biomarkers, liquid biopsy

## Abstract

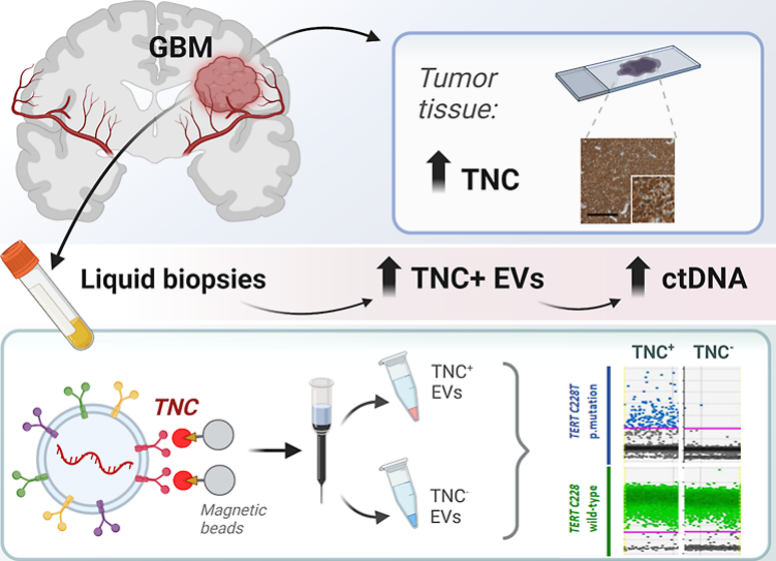

Extracellular vesicles (EVs) act as carriers of biological information from tumors to the bloodstream, enabling the detection of circulating tumor material and tracking of disease progression. This is particularly crucial in glioblastoma, a highly aggressive and heterogeneous tumor that is challenging to monitor. Using imaging flow cytometry (IFCM), we conducted an immunophenotyping analysis of eight glioma-associated antigens and tetraspanins in plasma EVs from 37 newly diagnosed glioblastoma patients (pre- and post-surgery), 11 matched individuals with recurrent glioblastoma, and 22 healthy donors (HD). Tenascin-C (TNC) positive EVs displayed the strongest differences in newly diagnosed and recurrent glioblastoma patients, when compared to non-tumor subjects. Among dual-positive subpopulations, TNC^+^/CD9^+^ EVs were the most elevated in newly diagnosed (FC = 7.6, *p* <0.0001, AUC = 81%) and recurrent patients (FC = 16.5, *p* <0.0001; AUC = 90%) than HD. In comparison with other CNS tumors (*n* = 25), this subpopulation was also 34.5-fold higher in glioblastoma than in meningioma cases (*p* <0.01). Additionally, TNC^+^/CD9^+^ EV levels were 3.3-fold elevated in cerebrospinal fluid from glioblastoma patients (*n* = 6) than controls (*p* <0.05). Aberrant TNC levels were further observed in glioblastoma EVs from different sources and purified via different methods. Immunohistochemical analysis revealed high levels of TNC in tumor tissues. Spatial transcriptomic analysis indicated a TNC overexpression in malignant cell populations of glioblastoma resections, particularly in cells with mesenchymal-like signatures and chromosomal aberrations. Lastly, we purified TNC^+^ EVs from plasma of 21 glioblastoma patients by magnetic sorting and detected the oncogenic mutation TERT*C228T by droplet digital PCR. The mutant allele frequency was higher in TNC^+^ EVs *vs* TNC-negative EVs (FC = 32, *p* <0.001), total EVs (FC = 5.3, *p* <0.001) or cell-free DNA (FC = 5.3, *p* <0.01). In conclusion, circulating TNC^+^ EVs may have potential as clinical biomarkers in glioblastoma, and their purification could improve the identification of tumor-specific mutations in liquid biopsies.

Glioblastoma is the most common and aggressive type of brain tumor.^1^ The diagnosis is established from tissue specimens obtained from biopsy or resection using histopathology, immunohistochemistry and increasingly molecular analyses (i.e., MGMT methylation status). However, glioblastoma tumors are highly heterogeneous, containing cell subpopulations with different transcriptional and epigenetic profiles, which hampers the subclassification of the whole tumor cell composition and therapeutic approaches.^1,2^ Considering the tumor heterogeneity, in addition to the invasiveness of tissue acquisition and surgical risks, it is imperative to identify clinical biomarkers for noninvasive monitoring of tumor progression during treatment, and also to obtain comprehensive preoperative tumor-specific information from liquid biopsies. In this respect, circulating extracellular vesicles (EVs) have gained attention as potential candidates.^3^ EVs are a heterogeneous group of nanovesicles released from cells, being the small EVs (approximately 80–200 nm sized) the most investigated and characterized in the field.^3−5^ EVs transport biological information from their original cells into the microenvironment and bloodstream, mediating short and long-distance intercellular communication. Therefore, EVs might enable noninvasive detection of tumor information and allow disease monitoring.^4,5^

Glioblastoma cells, as well as cells from the tumor microenvironment (TME), release high amounts of EVs, resulting in elevated EV levels in peripheral blood of glioblastoma patients.^6−8^ These tumor-derived EVs carry glioma-associated molecules,^5^ such as DNA copies reflecting the tumor methylation signatures and relevant oncogenic mutations in *TERT*, *EGFR, CDK2NA/B* and other genes.^8−12^ On the protein level, the expression of CD9, CD63 and CD81 tetraspanins, which are considered classical bona fide EV markers, may point toward to the cellular composition within the tumor (e.g., CD63 for immune cells) and tumor biological features (e.g., CD9 indicating immune regulatory mechanisms and tumor cell invasion),^13,14^ besides showing differential levels in glioblastoma-EVs.^7,8^ Additionally, high-throughput studies have identified various antigens with variable concentrations in EVs from patients with brain tumors. These include Tenascin-C (TNC), Integrin beta-1 (ITGB1, also known as CD29), CD44, Profilin-1 (PFN1), Secreted protein acidic and rich in cysteine (SPARC), Glycoprotein NMB (GPNMB), CD133 (also known as PROM1), and HLA-DR/DQ/DP.^9,15−29^

Despite their obvious potential as biomarkers, there is still a lack of studies evaluating these proteins in the different tetraspanin-positive subpopulations from plasma EVs, and determining their clinical usefulness. A major challenge toward this goal is the need to specifically separate the very small fraction of truly tumor-derived EVs (tEVs) from the bulk of background EVs constantly physiologically produced by all cells. Different strategies have been investigated to purify specific EV subpopulations from bulk samples, in order to address the limitations that prevent the translational use and analysis of EV cargo with sufficient specificity and sensitivity.^30,31^ However, reproducibility and efficacy of EV-DNA analysis are still insufficient. In this context, we performed a phenotyping analysis of the eight aforementioned antigens on tetraspanin-positive EVs to determine whether double-positive EV subpopulations may provide an incremental improvement for specificity of EV subgroup analysis. Among these, we found TNC on EVs to be a promising biomarker for glioblastoma. We further investigated TNC RNA and protein levels in glioblastoma cells, and identified TNC^+^ EVs as tEVs. The enrichment for this subpopulation was additionally associated with a detection improvement of a relevant tumor-derived mutation in the *TERT* gene promoter.

## Results

### Circulating TNC^+^ EVs are Elevated in Newly Diagnosed and Recurrent Glioblastoma, When Compared to Healthy Donors and Postoperative Subjects

Demographic and clinical data from different cohorts of patients enrolled in this study are described in Supporting Information Table S1. Collected EV samples were characterized in accordance with the current MISEV guidelines,^32^ and experimental descriptions were reported in EV-TRACK (EV-METRIC values of 63% for plasma EVs, and 57% for EVs isolated from cerebrospinal fluid [CSF] and from tissue resections; accession EV240008).^33^

**Figure 1:**
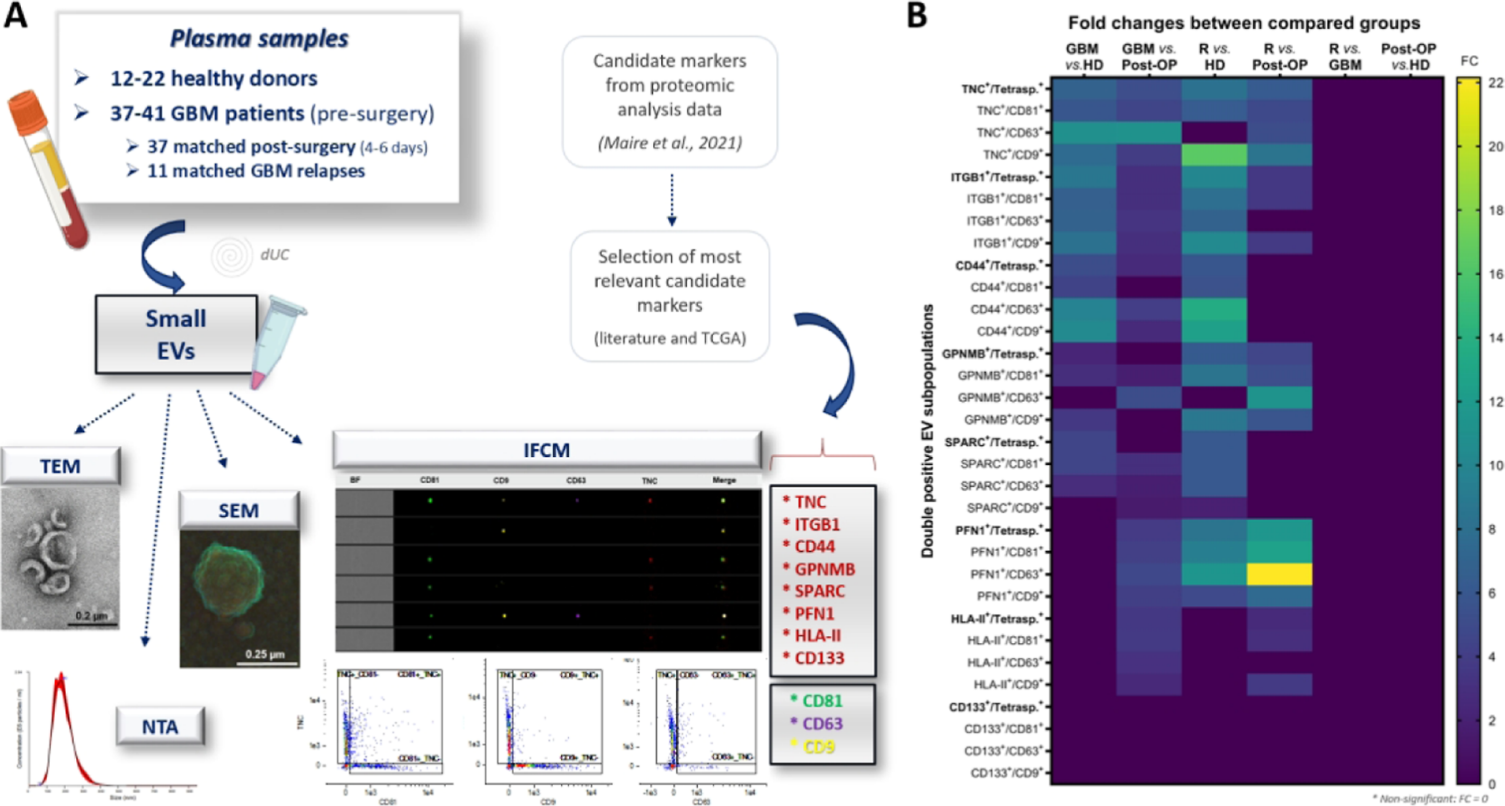
Plasma cohort and EV phenotyping overview. (A) EV phenotyping workflow showing cohort description and strategy of antigen selection for biomarker investigation by Imaging flow cytometry (IFCM). The candidate antigens TNC, ITGB1, CD44, GPNMB, SPARC, PFN1, HLA-DR/DQ/DP and CD133 (previously highlighted by our group),^9^ as well as classical EV markers (the tetraspanins CD81, CD63 and CD9) were selected for investigation, where the levels per milliliter of plasma of double-positive EVs (for each candidate marker and each tetraspanin) were compared among the groups. Figure made in BioRender.com. (B) Heatmap with significant fold changes (FC) when comparing the median levels of double positive EV subpopulations between groups (healthy donors (HD), newly diagnosed glioblastoma patients (GBM), postoperative (post-OP), and GBM relapses). Their main statistical analyses are shown in Figure 2. Nonsignificant results are plotted with FC equivalent to zero. TNC^+^ EVs (independently of the tetraspanins) are the most significantly increased in glioblastoma patients (newly diagnosed and relapses), in comparison to HD and post-OP subjects.

Plasma EVs showed the expected cup-shaped morphology, as visualized by TEM (Figure 1A), and NTA size mode range between 80 and 200 nm. As for tetraspanins, plasma EVs from all analyzed groups were mainly positive for CD9, followed by CD81 and CD63 (Supporting Information Table S1). All median concentrations per milliliter of plasma obtained by NTA and by IFCM (including all evaluated EV subpopulations), as well as the fold changes (FC) and p values for all comparison analysis are listed in Supporting Information Table S2.

**Figure 2:**
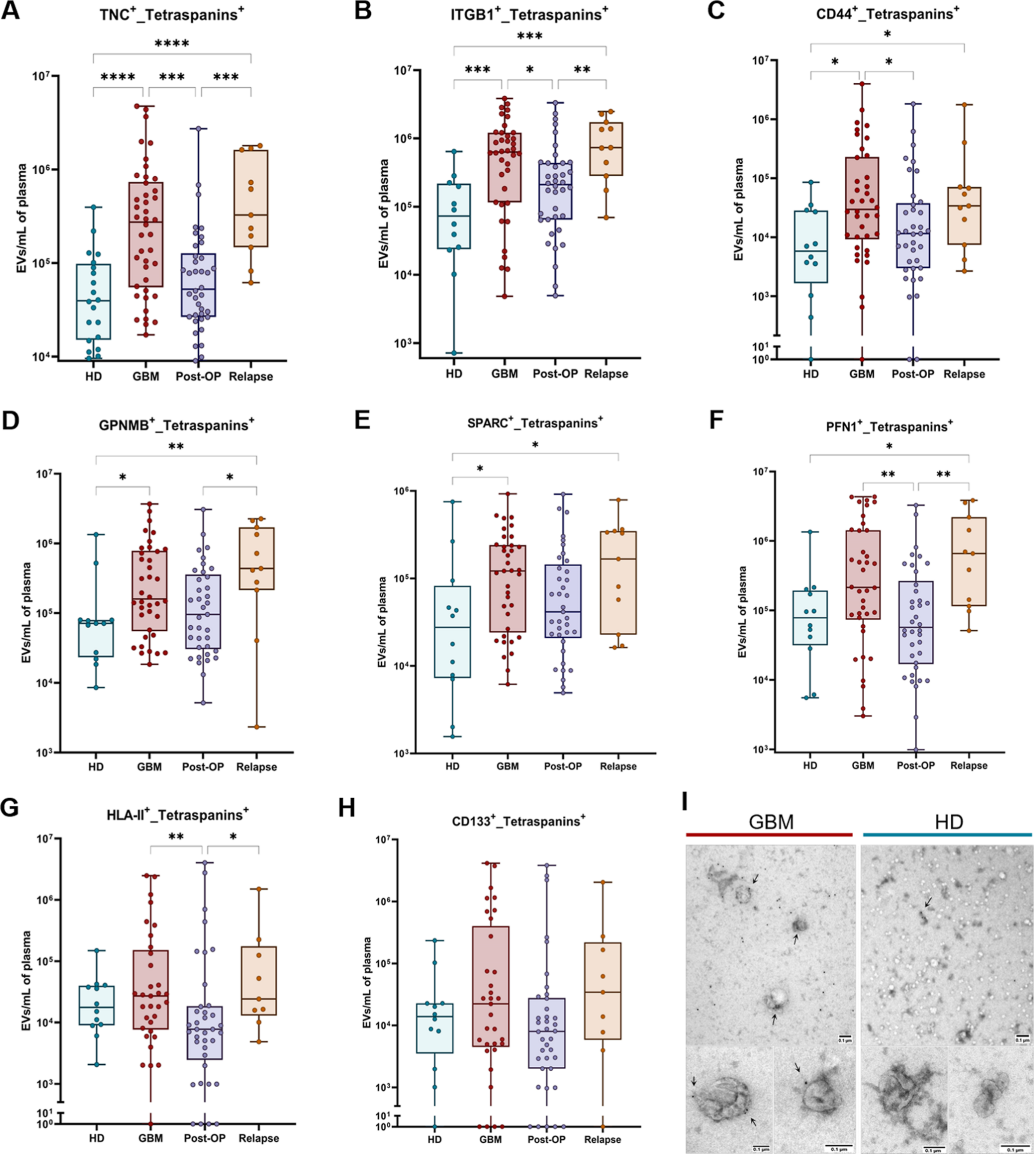
EV phenotyping analyses among the groups. (A–H) Plasma levels of EVs (positive at least for one of the tetraspanins) containing each of the selected candidate markers for investigation in HD (green), glioblastoma (GBM, red), post-OP (blue), and relapses (orange). Medians were compared between groups by Kruskal–Wallis analysis with correction for false discovery rate (FDR). Their respective FC values are shown in Figure 1B. The TNC^+^ EVs (A) were differentially expressed in most of compared groups and had the strongest significances (higher FC and lower p values), followed by ITGB1 (B). (I) Immunogold staining for TNC protein in plasma EVs of a glioblastoma patient (left) and a healthy donor (right). Obvious differences are observed between two immunostainings, where gold particles (black dots) can be visualized in plasma EVs from glioblastoma (white arrows). * = *p* < 0.05, ** = *p* < 0.01, *** = *p* < 0.001, **** = *p* < 0.0001. Nonsignificant associations are not specifically marked.

In our first approach, we evaluated EVs regarding their surface levels of glioma associated markers (Figure 1A), which were chosen according to a previous proteomic screening,^9^ and to their known differential expression in glioblastoma, according to the literature^9,15−29^ and TCGA data^34^ (Supporting Information Figure S1A–G). These eight candidate antigens (TNC, ITGB1, CD44, GPNMB, SPARC, PFN1, HLA-DR/DQ/DP and CD133) were herein immunophenotyped on EVs by IFCM, in combination with tetraspanins (CD81, CD63 and CD9), and quantified as double-positive EV subpopulations in plasma samples from 22 healthy donors (HD), 41 newly diagnosed glioblastoma (GBM) patients, 37 matched postoperative (post-OP) and 11 matched recurrent patients (relapse GBM). We found that most of the analyzed double-positive EV populations were significantly elevated in glioblastoma patients, with the exception of CD133^+^ EVs (Figure 1B and Supporting Information Table S2). TNC^+^ and ITGB1^+^ EV-populations (ITGB1 FC = 8.6 and 10 in newly diagnosed and relapse patients, respectively, versus HD; *p* < 0.001) presented different levels in most comparisons, regardless of the tetraspanin analyzed, followed by CD44, GPNMB and SPARC (Figures 1 and 2A–H and Supporting Information Table S2). Strong differences were also observed for PFN1^+^ EVs between post-OP and recurrent patients (FC = 22.2, *p* < 0.01). However, similarly to HLA-DR/DQ/DP, no significant results were found for any PFN1^+^ EV subpopulation between newly diagnosed and HD subjects, thus restricting the use of this antigen as a biomarker.

Given that TNC^+^ EVs exhibited the greatest disparities between newly diagnosed and recurrent glioblastoma when compared to HD (FC = 6.9 and 8.3, respectively; *p* < 0.0001) and to post-OP subjects (FC = 5.2 and 6.2, respectively; *p* < 0.001) (Figures 1B, 2A and Supporting Information Table S2), and considering TNC’s involvement in relevant pathways that are associated with glioblastoma progression (Supporting Information Figure S1H), we decided to focus on this antigen for our subsequent analysis. As expected, we detected elevated TNC signals in plasma EVs of a glioblastoma patient using immunogold TEM. In contrast, we observed only sporadic TNC signals in the EVs of an HD subject (Figure 2I). These findings corroborate the differential concentrations of TNC^+^ EVs in the blood circulation of glioblastoma patients. Taken together, TNC emerged as the most attractive antigen herein investigated, showing potential as a biomarker for glioblastoma.

### TNC^+^/CD9^+^ is the Main EV Subpopulation with Biomarker Utility in Glioblastoma

**Figure 3:**
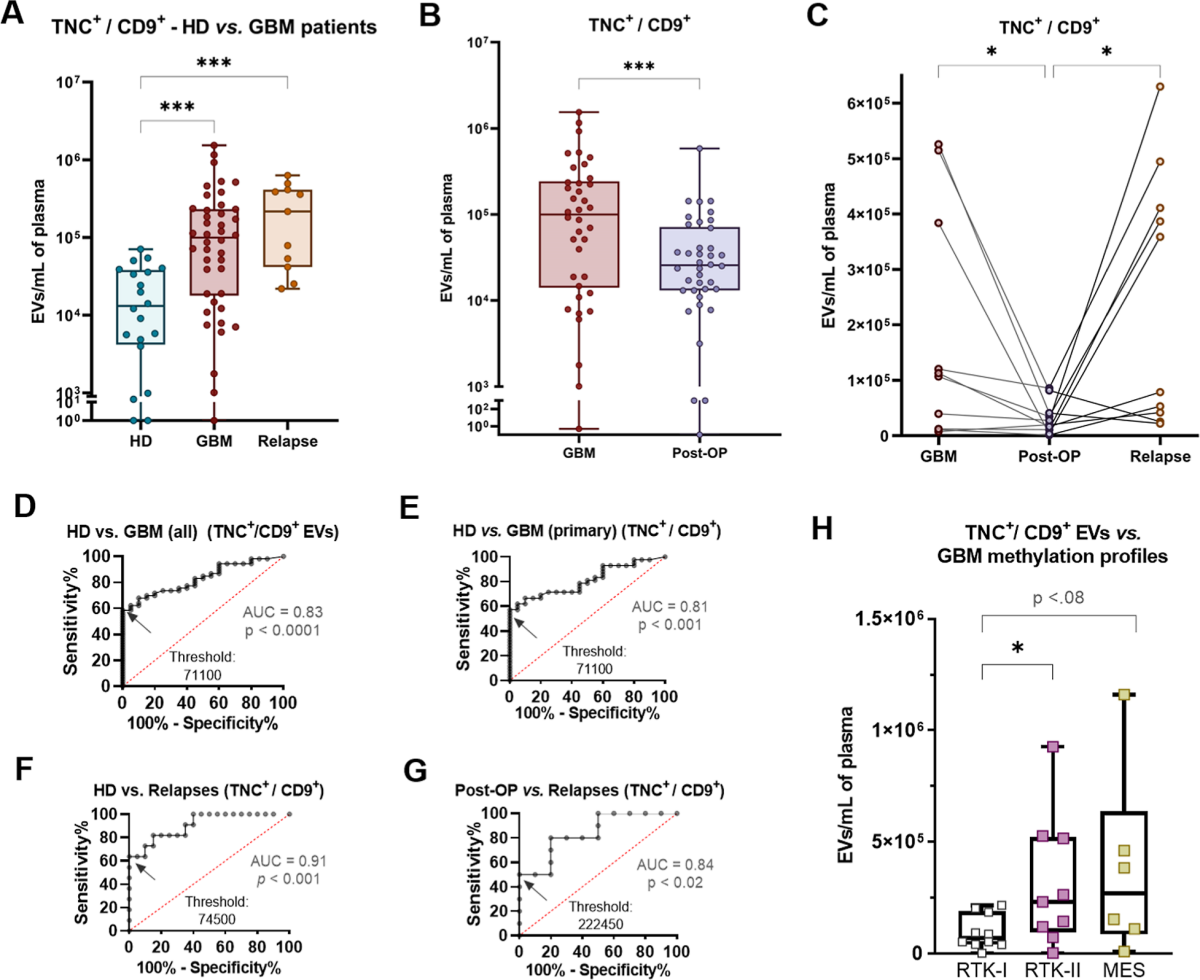
Differential levels of TNC^+^/CD9^+^ EVs in glioblastoma. (A) TNC^+^/CD9^+^ EVs are elevated in plasma of newly diagnosed (FC = 7.6) and recurrent (FC = 16.5) glioblastoma patients, in comparison to HD subjects. (B) A 3.9-fold drop is observed in a paired way for TNC^+^/CD9^+^ EVs after tumor removal, which (C) reincreased (FC = 8.4) in these same patients under tumor relapse. (D) ROC graph of TNC^+^/CD9^+^ EV levels, with area under curve (AUC) greater than 80% for discrimination between healthy donors and glioblastoma patients (newly diagnosed and recurrent together). (E) ROC curve of TNC^+^/CD9^+^ EVs, comparing HD and newly diagnosed glioblastoma, also with AUC greater than 80%. (F) ROC curve comparing HD and recurrent GBM, with the highest indicative results for TNC^+^/CD9^+^ EVs as clinical biomarkers, with AUC values greater than 91% (threshold 74,500 EVs for 100% specificity and 63% sensitivity). (G) ROC curve of TNC^+^/CD9^+^ EVs, with AUC of 84% for discrimination of post-OP subjects and recurrent glioblastoma. (H) TNC^+^/CD9^+^ EV levels in glioblastoma patients belonging to different methylation subgroups. Patients classified as RTK-I present 3.4-fold lower levels of TNC^+^/CD9^+^ EVs in plasma, in relation to patients with RTK-II subtype. * = *p* < 0.05, ** = *p* < 0.01, *** = *p* < 0.001, **** = *p* < 0.0001.

Among all dual-positive subpopulations assessed, TNC^+^/CD9^+^ EVs showed the highest significant disparities in most of the comparisons (Supporting Information Table S2). Their levels per milliliter of plasma were 7.6- and 16.5-fold higher in newly diagnosed and relapsed glioblastoma, respectively, when compared to HD (*p* < 0.0001) (Figure 3 A and Supporting Information Table S2). Significant results were also observed for TNC^+^/CD81^+^ in newly diagnosed and relapsed patients (FC = 5.9 and 6.4, respectively), as well as for TNC^+^/CD63^+^ (FC = 10.96 in newly diagnosed glioblastoma; *p* < 0.001) (Supporting Information Figure S2A,B and Supporting Information Table S2).

In paired analysis, glioblastoma patients exhibited a 3.9-fold decrease of TNC^+^/CD9^+^ EVs after tumor removal (*p* < 0.001) (Figure 3 B and Supporting Information Table S2). Additionally, there was a 4.5- and 11.1-fold decrease for TNC^+^/CD81^+^ (*p* < 0.001) and TNC^+^/CD63^+^ (*p* < 0.0001) EVs, respectively (Supporting Information Table S2). Paired TNC^+^ EV counts increased again upon tumor relapse, particularly in the case of TNC^+^/CD9^+^ (relapse FC = 8.4; *p* < 0.05) (Figure 3C and Supporting Information Table S2).

In order to evaluate if TNC^+^/CD9^+^ EVs could serve as clinical biomarkers, ROC analysis was performed. The results showed an area under curve (AUC) value of 83% (*p* < 0.0001) for discrimination between nontumor subjects (HD) and glioblastoma patients (newly diagnosed and recurrent together) (Figure 3 D). Specifically, TNC^+^/CD9^+^ EV concentrations significantly differentiated HD from newly diagnosed (AUC = 81%, *p* < 0.001) and especially from recurrent patients (AUC = 91%, *p* < 0.001) (Figure 3E,F). In the last case, TNC^+^/CD9^+^ EV levels higher than 74,500 per mL of plasma presented a discrimination between both analyzed groups with 100% specificity (thus equal or higher values not detected in HD) and 63% sensitivity. Possible deviations in the threshold values might occur between samples, volumes and operators. Significant values were also observed for this EV subpopulation when comparing post-OP with recurrent patients (AUC = 84%, *p* < 0.05), suggesting its predictive utility for tumor relapse (Figure 3G). Additional ROC differences between HD and glioblastoma patients were also observed for other TNC^+^ EVs populations (Supporting Information Figure S2C).

In regard to clinical parameters, TNC^+^/CD9^+^ EV counts also differed among glioblastoma methylation subtypes, with 3.34-fold lower levels in patients with the RTK-I epigenetic subtype compared to RTK-II (*p* < 0.05). A trend of 3.9-fold lower levels for TNC^+^/CD9^+^ EVs in RTK-I glioblastoma patients was also observed when compared with the mesenchymal (MES) subtype (*p* < 0.08) (Figure 3H). This finding raises the possibility to use EVs as preoperative informative agents for molecular subclassification. Meanwhile, TNC^+^/CD81^+^ EVs correlated with the peritumoral fluid-attenuated inversion recovery (FLAIR) hyperintensity (Spearman *r* = 0.144, *p* < 0.05), obtained via MRI (Supporting Information Figure S2D).

**Figure 4:**
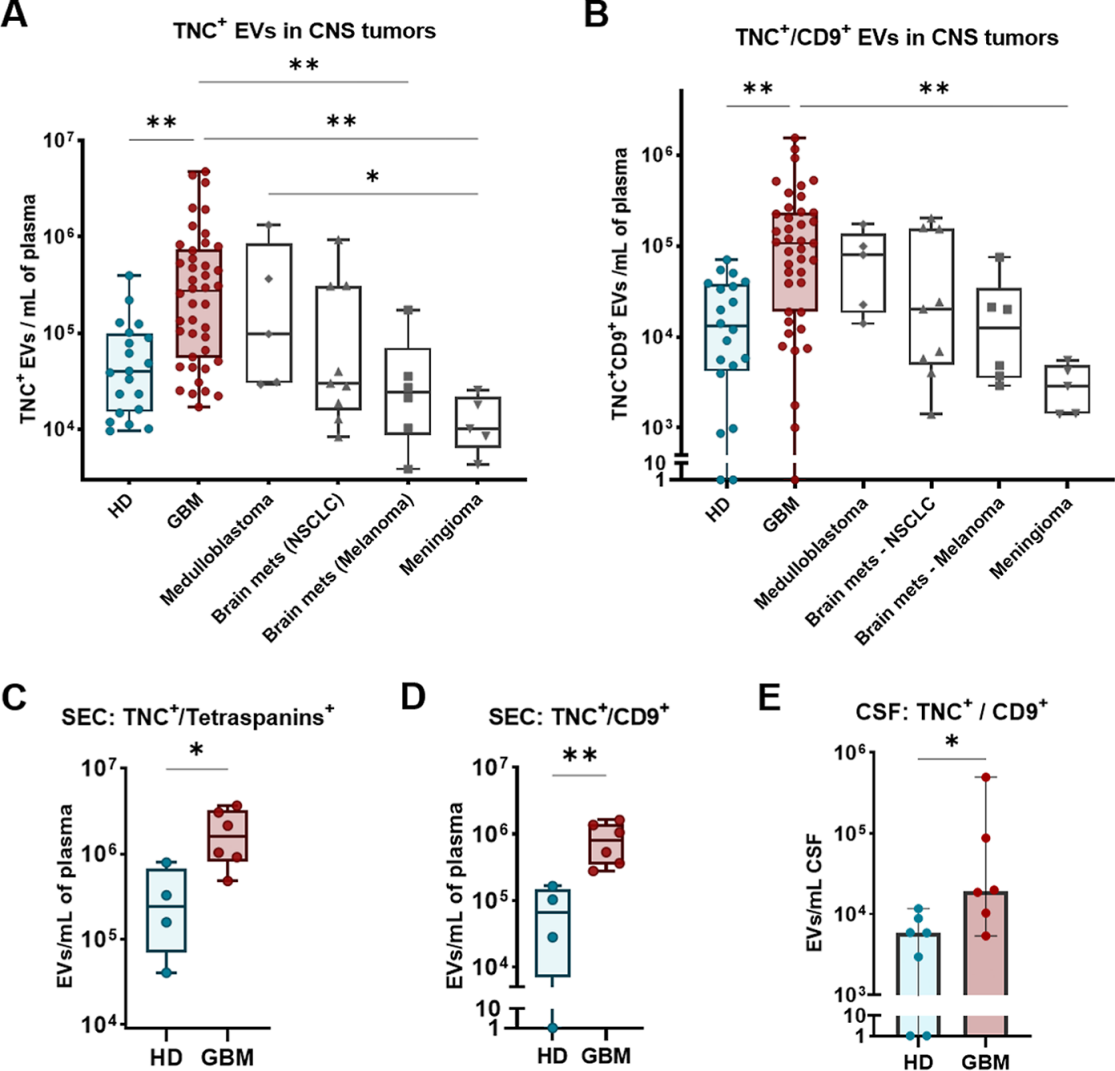
Differential levels of TNC^+^ and TNC^+^/CD9^+^ EVs in glioblastoma under different experimental conditions. (A) TNC^+^ EVs in comparison with other CNS malignancies (medulloblastoma, brain metastasis from NSCLC and from melanoma, and meningioma). Glioblastoma remains with the highest levels of TNC^+^ EVs per mL of plasma, also being 27.2- and 11.3-fold elevated than meningioma and brain metastatic melanoma, respectively. TNC^+^ EVs from medulloblastoma cases were also significantly higher than meningioma (FC = 9.7). (B) TNC^+^/CD9^+^ EVs in CNS malignancies. Glioblastoma remains with the highest levels of TNC^+^/CD9^+^ EVs per mL of plasma, also significantly elevated than meningioma cases (FC = 34.5). (C) TNC levels per mL of plasma on EVs isolated by size exclusion chromatography (SEC), which remained significantly increased in glioblastoma patients than in HD subjects (FC = 6.52). (D) TNC^+^/CD9^+^ levels per mL of plasma on EVs isolated by SEC, which remained significantly increased in glioblastoma patients than in HD subjects (FC = 12.05). (E) TNC^+^/CD9^+^ EVs were also significantly higher in cerebrospinal fluid (CSF) from glioblastoma patients, when compared to CSF from controls (suffering from normal pressure hydrocephaly) (FC = 3.3). * = *p* < 0.05, ** = *p* < 0.01.

A comparison with four other CNS malignancies (brain metastasis from NSCLC and melanoma, medulloblastoma and meningioma) revealed that plasma levels of TNC^+^ EVs were most elevated in the glioblastoma patients. These levels were significantly higher than those in patients with brain metastasis from melanoma (FC = 11.3, *p* < 0.01) and medulloblastoma (FC = 9.7, *p* < 0.05). Statistically significant differences were also observed for TNC^+^ EVs (FC = 27.2, *p* < 0.01) and the subpopulation TNC^+^/CD9^+^ (FC = 34.5, *p* < 0.01) when compared to meningioma patients (Figure 4A,B).

Additionally, to validate our results in EV samples isolated with a second method, we evaluated TNC levels on EVs purified from plasma of a parallel glioblastoma cohort using size exclusion chromatography (SEC), which is an easy and gentle isolation method that can be performed from low sample volumes. In this instance, elevated TNC^+^ EV levels remained statistically significant in glioblastoma patients when compared to HD, particularly in the case of TNC^+^/CD9^+^ EVs (FC = 12.05, *p* < 0.01) (Figure 4C,D). These findings suggest that TNC^+^/CD9^+^ EVs may serve as an optimal biomarker for discrimination between glioblastoma and healthy individuals. Furthermore, we observed a 3.3-fold increase in the levels of TNC^+^/CD9^+^ EVs in cerebrospinal fluid (CSF) samples of glioblastoma patients (newly diagnosed and recurrent cases were analyzed together), in comparison to controls (subjects with normal pressure hydrocephalus) (*p* < 0.05) (Figure 4E). These findings provide further support for the use of TNC^+^/CD9^+^ EVs as clinical biomarkers for glioblastoma, including those derived from a range of conditions and sample sources.

### TNC Levels are Elevated in Tumor Cells and in EVs from Glioblastoma Tissues

Intensities of TNC protein expression were evaluated in 30 paired glioblastoma tissues using immunohistochemistry (IHC). Digital histo-scores (DH-score) were calculated (Supporting Information Figure S3 and Supporting Information Table S3). The majority of glioblastoma tissues exhibited a strong and diffuse expression of TNC protein (86.7% with DH-score >100; Supporting Information Figure S3A–I,M), with the exception of four cases who had DH-scores lower than 100 (Supporting Information Figure S3M and Supporting Information Table S3). No evaluated sample presented null DH-score values for TNC. In parallel, TNC intensities in non-neoplastic tissues from epileptic patients (without structural alterations), where TNC levels are also known to be higher than in homeostasis,^35,36^ were most prominent in the white matter (as expected for a non-neuronal marker). In epileptic brain cortex, TNC immunostaining signal was predominantly faint (Supporting Information Figure S3J–L).

As we hypothesized that the majority of TNC^+^ EVs observed in the peripheral blood of glioblastoma patients are released from TNC^high^ tumor cells, we sought to also quantify TNC in EVs derived from tumor tissues. EVs were isolated from 12 glioblastoma resections with high TNC DH-score (>200, as exemplified in Supporting Information Figure S3A–F,M) and/or with high plasma TNC^+^ EV levels (>5 × 10^5^ EVs per mL of plasma) by density gradient dUC, and analyzed by IFCM (Supporting Information Table S3). TNC^+^ EV counts from glioblastoma-tissue resections (median = 2.59 × 10^8^ per milligram of tissue) were 240-fold higher than in matched plasma (Supporting Information Figure S3N), adding proof that tumor cells are the main source of elevated release of TNC^+^ EVs. Furthermore, high levels of TNC on the EV membrane were also detected in tetraspanins^+^ EV subpopulations from a glioblastoma tissue, by dSTORM analysis (Supporting Information Figure S3O). Additional dSTORM single-sample characterizations are shown in Supporting Information Figure S4 and are consistent with the results of our IFCM analysis (Supporting Information Table S3).

### TNC mRNA is Enriched in Specific Glioblastoma Clusters Corresponding to Malignant Signatures

**Figure 5:**
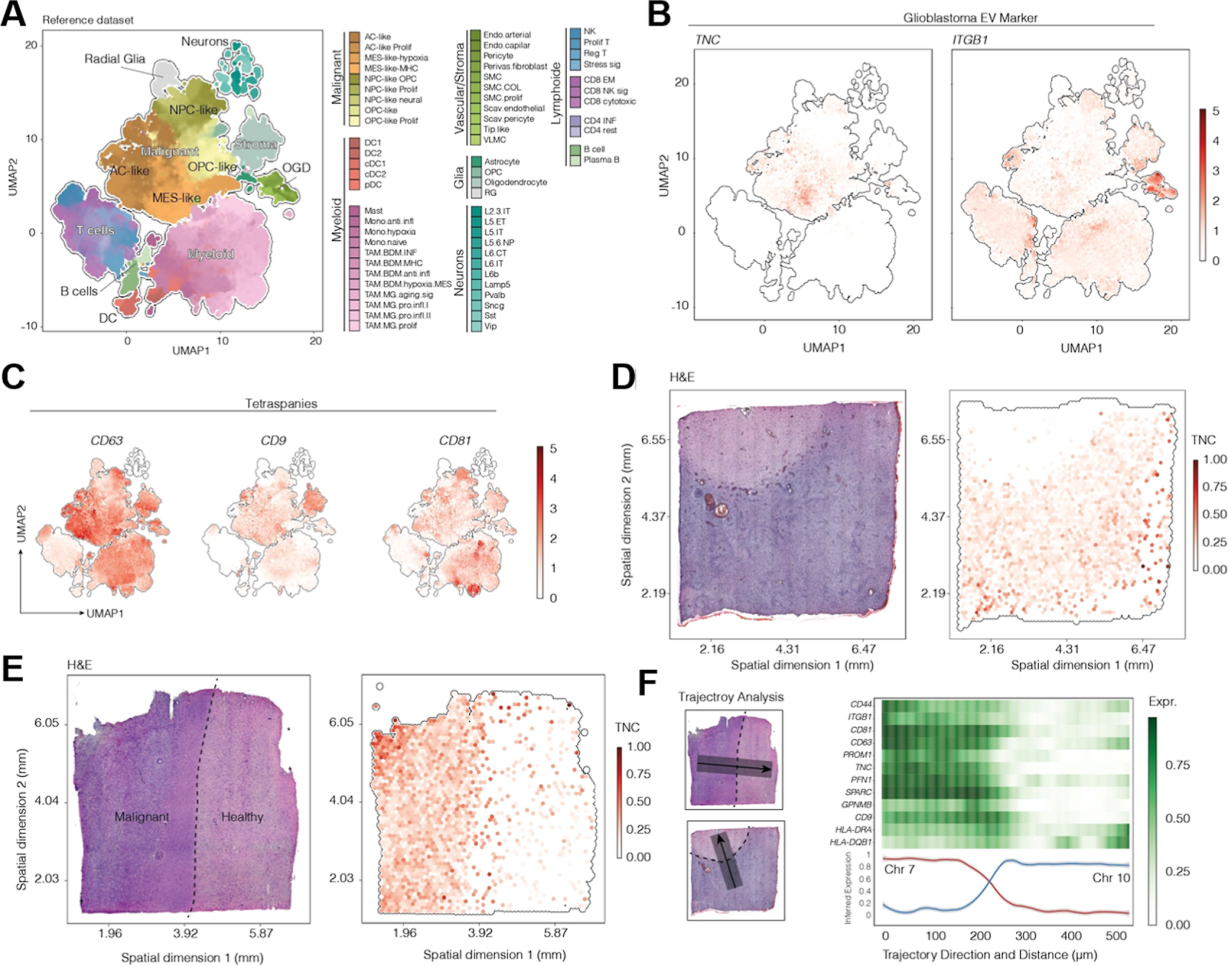
TNC mRNA expression in glioblastoma tissue by spatial transcriptomic analysis. Expression of investigated markers in cell populations of glioblastoma tissue, as classified by spatial transcriptomics analysis. (A) Leveraging the glioblastoma reference single cell data set (GBMap) to explose distinct transcriptional signatures within the tumor tissue, of which the genuine malignant area is represented by glioblastoma subtypes classified as neural progenitor-like (NPC), mesenchymal-like (MES), astrocyte cell-like (AC) and oligodendrocyte progenitor cell-like (OPC). (B) RNA expression of TNC and ITGB1 markers within the tumor tissue. TNC expression mainly corresponds to the glioblastoma malignant signature areas, in contrast to ITGB1. (C) Also in contrast to TNC, and similarly to the other evaluated antigens, the tetraspanins (CD63, CD9 and CD81) are nonspecifically expressed throughout the tumor tissue. (D, E) As visualized by H&E staining, TNC is overexpressed in the malignant area of the tumor tissue. (F) Following the trajectory direction from the malignant to the peritumoral zone, TNC and most of the investigated markers are exclusively overexpressed in glioblastoma cells, which also colocalize with oncogenic CNVs, such as gain in chromosomes 7 and loss in chromosome 10.

We evaluated TNC mRNA levels leveraging the glioblastoma reference single cell data set (GBMap),^37,38^ to explore its specific expression pattern in different cell populations within the heterogeneous tumor (Figure 5A–F). For comparison, spatial mRNA levels of ITGB1 and tetraspanins (CD81, CD9 and CD63) (Figure 5A–C,F), as well as of CD44, CD133 (PROM1), PFN1, SPARC, GPNMB, HLA-DRA and HLA-DQB1 (Figures 5F and Supporting Information S5) were also assessed.

We found that, in exception of TNC, the transcriptional expression of tetraspanins and other herein analyzed antigens were not exclusively localized to malignant cells (Figures 5A,B and Supporting Information S5). In contrast, TNC was specifically overexpressed in malignant cell populations, particularly in tumor cells with mesenchymal-like (MES-), astrocyte cell-like (AC−) and oligodendrocyte progenitor cell-like (OPC−) subtypes (Figure 5A,B). Based on the observation that EV markers showed only low expression in the neuronal cell populations (Figures 5A–C and Supporting Information S5), we hypothesized that they are mainly expressed by the tumor microenvironment and cells that are tumor associated. To test this hypothesis, we evaluated markers’ expression in spatially resolved transcriptomic data from the Visium platform. We observed an enriched expression of TNC in the malignant tumor compartment compared to the normal brain regions in the periphery (Figure 5D,E). Spatial trajectory analysis confirmed a sharp expression loss of TNC and of most evaluated markers at the tumor border, which significantly correlate with the chromosomal alterations Chr7 gain and Chr10 loss (Figure 5F). These findings further reinforced the association between TNC expression with glioblastoma cells and molecular signatures of malignant progression.

### TNC^+^ EVs Enrichment Improves TERT*C228T Oncomutation Detection

To determine the suitability of TNC protein to enrich tumor-derived EVs (tEVs) from the circulation, we performed MACS sorting of TNC^+^ EVs from plasma samples of glioblastoma patients (Supporting Information Figure S6A). The binding success between EVs and 50 nm magnetic beads was visualized by SEM (Supporting Information Figure S6B), and the TNC enrichment was confirmed by FACS (Supporting Information Figure S6C), and both for glioblastoma and a healthy control by IFCM (Supporting Information Figure S6D). The sorted TNC^+^ and TNC^–^ EV fractions were evaluated by droplet digital PCR (ddPCR), in order to compare allele frequencies of TERT*C228T, the most common mutation in glioblastoma patients. It is located in the promoter of telomerase gene in tumor cells and absent in non-neoplastic cells, thus being a mutation of choice for biomarker purposes in glioblastoma.^10,39^ In our ddPCR data, all samples with less than 30 positive droplets for wild-type *TERT* promoter copies were excluded from analysis, and all results with 0 or 1 positive droplet for the *TERT C228T* were considered as negative. All nontemplate controls and negative controls (samples known to lack the mutation) showed negative results (data not shown).

**Figure 6:**
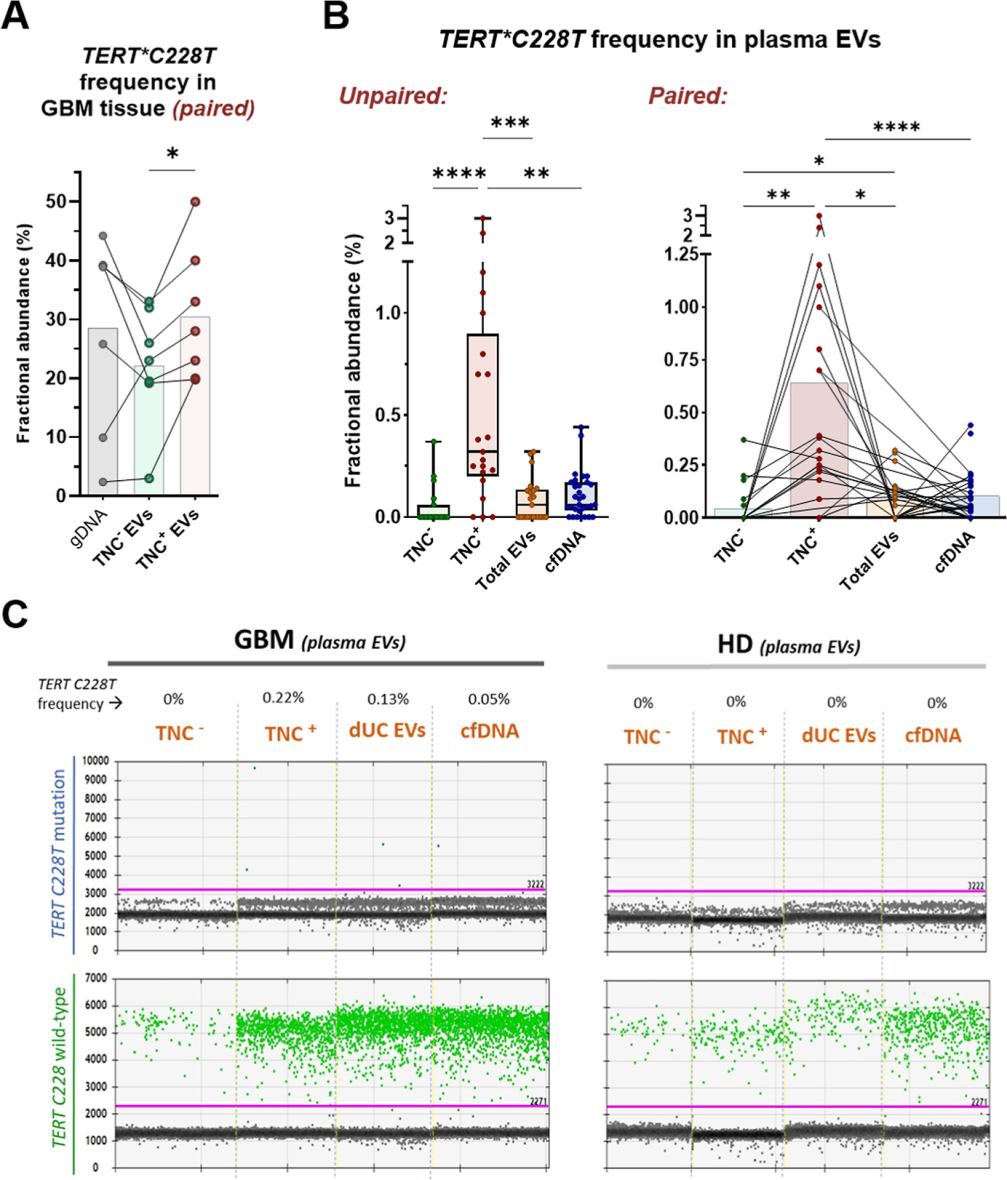
Tumor-derived DNA analysis in TNC^+^ and TNC^–^ EV fractions. (A) Paired analysis of the TERT*C228T tumor mutation frequencies in gDNA and TNC-sorted EV-DNA samples obtained from glioblastoma-tissue resections (*n* = 7), by droplet digital PCR (ddPCR). The samples are from the cohort of tissue-EVs previously analyzed by IFCM (see Supporting Information Figure S3) and are from tumors carrying the *TERT* mutation. TNC^–^ EVs (green) seem to carry lower or similar mutation frequencies than genomic DNA (gray) (nonsignificant differences), in addition to and 1.22-fold lower TERT*C228T frequencies than TNC^+^ EVs (red) (*p* < 0.05), when analyzed by Student *t*-test. Tissue-derived TNC^+^ EVs present significant higher frequencies of mutation amplicons (8.3% more abundant, in average) than their paired TNC^–^ fraction. (B) TERT*C228T frequencies in plasma samples. Left panel: unpaired analysis (Kruskal–Wallis with FDR correction), where circulating TNC^+^ EVs are shown with significantly higher frequencies of tumor mutation than EVs lacking TNC (FC = 32), total EVs (FC = 5.3) and cfDNA (FC = 5.3). Right panel: paired analysis of the same cohort (Wilcoxon test). Differences remained significant in paired comparisons, in addition to a 6-fold decrease in TNC- EVs than total EVs. (C) Examples of ddPCR plots for analysis of TERT*C228T tumor mutation in TNC^–^ EVs, TNC^+^ EVs, total EVs and bulk cfDNA from plasma samples. Droplets carrying mutation amplicons are shown in blue (upper plots) whereas wild-type copies in green (green droplets, below). The analyzed *TERT* mutation is mainly observed in DNA of TNC^+^ EVs. On the right side, a similar analysis was performed with HD samples to show the absence of unspecific amplifications. # = *p* < 0.08, * = *p* < 0.05, ** = *p* < 0.01, *** = *p* < 0.001, **** = *p* < 0.0001.

First, we evaluated the TNC^–^ and TNC^+^ EVs purified from 7 glioblastoma-tissue resections (samples of paired tissue-derived EVs analyzed by IFCM) containing the *TERT C228T* mutation, according to ddPCR results of tissue genomic DNA (Figure 6A). It is important to note that total EVs isolated from tissue are already naturally enriched in tEVs, which might include not only an elevated amount of TNC^+^ EVs, but also of tEVs lacking TNC (i.e., released from TNC^low^ tumor cells). In addition, nontumor cells in the TME may also contribute with TNC^+^ EVs to the total isolated EV pool. Despite that, TNC^–^ tEVs in the tumor core are still abundant enough to provide tumor-derived DNA that will be efficiently detected by ddPCR. Nevertheless, we observed an average increase of 8.3% for the TERT*C228T mutation frequency in the TNC^+^ EVs, when compared to TNC^–^ EVs (FC = 1.22, *p* < 0.05) (Figure 6A). Interestingly, the mutation frequencies detected in tissue-derived TNC^+^ EVs tended to be similar or even higher than in bulk genomic DNA from the whole tissue, although these differences did not reach significance.

Next, we analyzed the frequencies of TERT*C228T in TNC^–^ and TNC^+^ EV fractions from plasma samples, where tumor-derived EVs are rare and difficult to detect. For comparison purposes, we also included their paired total EVs (directly after dUC) and bulk plasma (cfDNA) samples for analysis (Figure 6B,C). Despite the expected low mutation frequencies observed in these samples, we found that the DNA carried by TNC^+^ EVs contained 32-times higher frequencies of the *TERT* oncomutation than EVs lacking TNC (*p* < 0.0001), and 5.3-fold higher frequencies compared to both bulk total EVs (*p* < 0.001) and bulk cfDNA (*p* < 0.01) (Figure 6B, **left panel**). In paired analysis, the differences remained significant (*p* < 0.01, *p* < 0.5 and *p* < 0.0001, respectively), in addition to a 6-fold decrease in TNC^–^ EVs when compared to total EVs (*p* < 0.05) (Figure 6B, **right panel**). Examples of ddPCR plots for analysis of TERT*C228T in plasma samples of a glioblastoma patient and a HD (as control, without mutation amplification) are shown in Figure 6C.

These results indicate that a significant proportion of circulating EVs in glioblastoma patients is derived from the tumor cells and suggest that their enrichment via magnetic sorting for TNC can facilitate a more sensitive detection of tumor-specific genetic alterations.

## Discussion

Glioblastoma is the most common malignant brain tumor in adults and is highly aggressive and molecularly heterogeneous.^1^ Due to its difficult accessibility, high therapeutic resistance and short-term survival rates, it is desirable to improve patients’ diagnosis, classification and monitoring through a noninvasive method in addition to imaging. In this context, the analysis of circulating tumor markers on EVs from liquid biopsies has gained extensive attention in recent years.^5,6^

In this study, we conducted a phenotypic analysis of eight glioma-associated antigens that were previously identified in a protein screen by our group.^9^ We analyzed these proteins in plasma EVs from glioblastoma patients and controls, in combination with classical EV markers. The majority of the double-positive EV subpopulations were confirmed as elevated in glioblastoma. Among them, TNC^+^ and ITGB1^+^ EVs showed significantly higher levels in newly diagnosed and recurrent glioblastoma patients compared to HD and post-OP subjects. However, ITGB1 was found to be nonspecifically expressed in glioblastoma tissue according to spatial transcriptomic analyses, despite the high ITGB1^+^ EV counts in glioblastoma patients, which is also supported by the literature,^9,25,26^ suggesting that much of this fraction is contributed by the TME. Furthermore, previous studies have also shown that CD44^+^, GPNMB^+^ and SPARC^+^ EVs are elevated in glioblastoma patient plasma.^9,20,25−27^ Interestingly, PFN1 exhibited the strongest differences between post-OP and relapses, hinting at a possible involvement of PFN1 in tumor resistance mechanisms. Aberrant expression and specific isoforms of PFN1 have been associated with tumor advanced stages, aggressiveness, progression and poor patient survival.^18,40−43^ However, the clinical utility of PFN1^+^ EVs as biomarkers might be restricted to the postoperative/progressive phase, due to its lack of significant differences between newly diagnosed glioblastoma and HD plasma samples.

By exploring TNC, whose expression has been associated with glioblastoma for a long time,^44^ we found that all TNC^+^ EV subpopulations were elevated in glioblastoma patients, both in newly diagnosed and recurrences. We confirmed the higher TNC signals in plasma EVs of a glioblastoma patient through immunogold EM images. Although newly diagnosed glioblastoma had the highest plasma fold changes for TNC^+^/CD63^+^ EVs than HD, weak or insignificant differences were found for this subpopulation in recurrent patients compared to HD and post-OP subjects. Possibly, the subpopulations of TNC^+^/CD63^+^ EVs may primarily originate from tumor-infiltrating immune cells,^45,46^ since CD63 has been shown to be upregulated in glioma-associated macrophages,^13,47^ and its mRNA was herein observed to colocalize with tumor-infiltrating myeloid cells. Meanwhile, TNC^+^/CD81^+^ plasma EVs showed the highest counts in all analyzed groups (tumor and nontumor) and the weakest (but still significant) fold changes in glioblastoma, aligning with our findings on TNC^+^/CD81^+^ levels in tissue-derived EVs and the unspecific spatial expression of CD81 in brain tissue (particularly from myeloid cells). Moreover, TNC^+^/CD81^+^ EVs correlated with FLAIR hyperintensity, which besides tumor cell infiltration, might also be indicative of peritumoral edema.^8,48^

Meanwhile, EVs from newly diagnosed and recurrent glioblastoma patients show the strongest differential levels of TNC^+^/CD9^+^, particularly when compared to HD subjects. Importantly, TNC^+^/CD9^+^ EVs significantly decreased in newly diagnosed glioblastoma patients after tumor removal and reincreased in the same individuals when the tumor recurred. The higher TNC abundance observed in recurrent patients could be a consequent response to radiotherapy.^49−51^ Also, when compared to the methylation-based subclassification, lower TNC^+^/CD9^+^ values emerged as indicative of the RTK-I epigenetic subtype. Therefore, the analysis of TNC^+^/CD9^+^ EVs holds promise for patient diagnosis, monitoring, predicting clinical subtypes, and also guiding therapeutic approaches. In ROC analysis, TNC^+^/CD9^+^ EVs exhibit the greatest sensitivity and specificity values for glioblastoma detection. Finally, the concentration of TNC^+^/CD9^+^ EVs was also significantly elevated in CSF samples of glioblastoma patients, although with a lower fold change than in plasma samples. This may be due to the biological characteristics of different body fluids and to the fact that TNC, as with a transient low-basal expression in CNS tissues,^52,53^ might also be detected in CSF from healthy individuals. Moreover, glioblastoma patients often exhibit blood–brain barrier (BBB) disruption, which, along with increased vascularization within the tumor region, might favor a higher release of tumor-derived EVs from the TME directly to the blood (what does not happen with the intact BBB in controls), resulting in higher proportions of TNC^+^/CD9^+^ EVs in their plasma compared to CSF. Nonetheless, the significant differences observed in EVs from both plasma (by UC and SEC) and CSF samples support the potential of TNC^+^/CD9^+^ EV quantifications as clinical biomarkers for glioblastoma, across different methods and liquid biopsy sources.

Spatial transcriptomics analysis confirmed that TNC mRNA overexpression specifically colocalizes with regions of high malignant cell content, particularly with areas of glioblastoma cells with MES-like signatures. Also, TNC expression mainly occurs in tumor areas that contain relevant and frequent chromosomal aberrations in glioma, such as gain of chromosome 7.^9,54^ This suggests that TNC is overexpressed in the genuine tumor cells, which is in agreement with our immunostaining results in glioblastoma tissue and previous immunohistochemical studies.^55^

Given that glioblastoma cells overexpress TNC and naturally release high amounts of EVs,^6,22,23^ and that an excess of brain-derived EVs in the blood circulation likely results from BBB disruption,^56^ it appears evident that the majority of TNC^+^ EVs in the blood plasma indicate a brain malignancy. Indeed, elevated levels of TNC were observed in EVs isolated from glioblastoma-tissue resections. Taken together, we hypothesize that TNC^+^ cells from the tumor region are primarily responsible for the excess of plasma TNC^+^ EVs observed in these patients, which could be suitable for translational purposes.

An additional goal of this study was to determine whether glioblastoma-associated markers carried by EVs could be used as a strategy to enrich tumor-derived EVs (tEVs) from plasma, allowing noninvasive molecular characterization of the tumor. Having identified TNC as the most promising circulating EV marker for this disease, we thus enriched tEVs via magnetic sorting of TNC and evaluated the presence of DNA copies carrying the TERT*C228T mutation. This mutation is found in approximately 70% of *IDH1*-wild-type glioblastoma and is linked to telomerase activation.^39,57^ Despite its clinical relevance, detecting TERT*C228T in liquid biopsies remains challenging due to its low frequency compared to other nontumor DNA copies in bulk plasma. The DNA of sorted TNC^+^ EVs was compared to paired fractions of TNC^–^ EVs, total EVs, and bulk cfDNA. Indeed, enrichment of TNC^+^ EVs significantly improved the detected frequencies of TERT*C228T, reinforcing that the majority of TNC^+^ EVs in plasma from glioblastoma patients are specifically released from tumor cells, unlike TNC^–^ EVs. Additionally, *TERT* mutated copies were 5-fold more frequent in TNC^+^ EVs than in bulk cfDNA, which is currently mainly used in liquid biopsy studies and may not be as informative due to its poor “signal to noise” ratio. This highlights TNC^+^ EVs as a better option to detect clinically relevant molecular alterations in glioblastoma from liquid biopsy.

TNC is an extracellular matrix component that is known to be involved in the progression of brain tumors.^24^ This glycoprotein is overexpressed in developmental stages and immature CNS cells, with significantly decreased levels in mature cells after birth.^58^ High TNC levels in adult CNS are associated with pathological conditions, such as neuro-inflammation, BBB disruption and brain tumors.^58^ TNC has been shown to be associated with immunosuppression, angiogenesis, proliferation and invasiveness in brain cancers,^22,23,59−63^ and in contrast to other malignancies (where it is mainly secreted from surrounding fibroblasts and endothelial cells), glioma cells are the main source of TNC rather than cells in the TME.^58^ Aberrant levels of TNC have also been observed in EVs from glioblastoma patients^22^ and of other cancer types.^64,65^ Yet, our study is the first to investigate both plasma and tissue levels of TNC^+^ EVs (I) stratified by tetraspanins, as double-positive EV subpopulations in glioblastoma patients from different time-points; (II) in relation to both mRNA and protein expressions of TNC in glioblastoma and nontumor brain cells; and (III) as source for enrichment of tumor-derived EVs. However, we also faced expected limitations regarding the final DNA yields after EV pulldowns, which prevented us from analyzing some paired samples. Nevertheless, with the increasing methodological refinements in the EV field and for the analysis of circulating tumor-derived DNA, addressing these technical limitations in the near future may allow the analysis of specific EV subpopulations that are informative of their cells of origin. Our results suggest an effective strategy for enriching a specific EV subpopulation in glioblastoma, offering a promising approach for purifying tumor-derived DNA from the majority of ’contaminant’ DNA found in circulating EVs. Further analysis enrolling other molecular entities (e.g., tumor-associated mRNAs, miRNAs, proteins), relevant tumor-derived mutations (e.g., EGFRviii; copy number variations in *PTEN* and *CDKN2A/B* genes) and different EV isolations methodologies, is encouraged to reach clinical translation. Comparative studies with other diseases are also needed to quantify TNC as biomarkers in a personalized manner, in patients suspected of glioblastoma or other specific conditions where this protein may play a role, in parallel with established clinical approaches and parameters.

In conclusion, this study demonstrates that TNC on EVs has potential as a biomarker for glioblastoma. Additionally, once optimized and standardized, quantifying TNC^+^ EVs could be implemented as a routine noninvasive option for monitoring tumor patients and establishing correlative parameters of future targeted therapies. The establishment of a method for TNC^+^ EV enrichment herein described provides a first step toward analyzing plasma EV content reflecting tumor biology.

## Conclusion

We identified combinations of surface antigens on EVs that are specifically increased in glioblastoma. Among these, we highlight TNC^+^ EVs, which have clinical potential as glioblastoma biomarkers and for purifying tumor-derived EVs. Analyzing TNC^+^ EV-DNA cargo has the potential to optimize downstream analyses in glioblastoma plasma samples for translational purposes.

## Material and Methods

### Patients’ Cohorts and Isolation of Extracellular Vesicles

The subjects enrolled in this study included glioblastoma patients and healthy donors from Germany, attended in University Medical Center Hamburg-Eppendorf, between 2019 and 2022. This study is in accordance to German federal laws and to the Declaration of Helsinki. Demographic and clinical data from our cohorts are detailed in Supporting Information Table S1. For glioblastoma patients, preoperative T1-and T2-weighted MRI axial images were analyzed using the cranial planning software Brainlab, in order to respectively measure tumor volume (in cm^3^) and peritumoral FLAIR hyperintensity, as previously described.^8^ EV characterizations are in accordance to the current MISEV guidelines^32^ and all relevant data concerning EV experimental cohorts were submitted to the EV-TRACK knowledgebase (accession number: EV240008).^33^

For biomarker investigation, peripheral blood samples were collected from 37 to 41 newly diagnosed glioblastoma patients (CNS WHO grade 4, *IDH1* wild-type) before surgery, 37 matched postoperative (post-OP) patients (4 to 6 days after surgery), 11 matched glioblastoma relapses and 12–20 healthy donors. A comparison cohort composed of meningioma (*n* = 5), medulloblastoma (*n* = 5), brain metastatic from NSCLC (*n* = 9) and from melanoma (*n* = 6) patients was also enrolled. Plasma fractions were obtained from whole blood (centrifuged at 1,000*g* for 7 min) and cleared to deplete the platelets (centrifuged at 2,000*g* for 10 min, 4 °C). Platelet removal was observed in these samples (*n* = 3) by IFCM phenotyping of CD41/CD61 markers in CD81^–^/CD9^+^ small particle populations (data not shown). Circulating EVs were obtained from platelet-poor plasma by differential ultracentrifugation (dUC). Briefly, 2–10 mL of cleared plasma were centrifuged at 10,000*g* for 40 min (4 °C), to deplete the large EVs. The supernatants were then submitted to ultracentrifugation at 100,000*g* for 70 min (4 °C) in polypropylene centrifugal tubes (Beckman Coulter, cat no. 361,625), using the fixed rotor MLA-50 (Beckman Coulter). Pellets of small EVs were resuspended in 0.22 μm-filtered PBS and immediately frozen at −80 °C until use. In addition to dUC-isolated EVs, a parallel plasma cohort of first-diagnosed glioblastoma (*n* = 5) and HD (*n* = 4) was also utilized for EV isolations by size exclusion chromatography (SEC), using a qEV2 Izon column (Izon Science), following manufacturer’s instructions. Briefly, 2 mL of PPP was loaded in SEC columns, and the eluted fraction containing purified EVs (8 mL after buffer volume) was collected, and concentrated using 100 kDa Amicon filters (UFC901024, Merck Millipore).

For some experiments, EVs from cerebrospinal fluid (CSF) and from glioblastoma-tissue resections were also included. CSF was obtained preoperatively via lumbar puncture or during the operation, from 6 glioblastoma patients (3 newly diagnosed, 3 recurrent; 83% male, median age 67.5 years old) and 7 controls (subjects with normal pressure hydrocephalus; 43% male, median age 77 years old). CSF samples (0.5–2 mL) were first cleared (1,000*g* for 7 min, followed by 2,000*g* for 10 min at 4 °C) and depleted of large-EVs (10,000*g* for 40 min, 4 °C). After, the cleared CSF samples were concentrated, using 300 kDa filters (Nanosep; centrifuged at 4,000*g* for 20–120 min at 4 °C) and resuspended in filtered PBS.

For tissue-derived EVs, 12 glioblastoma resections from newly diagnosed patients were selected (matched with plasma cohort, from patients who presented TNC^+^ EV levels higher than 5 × 10^5^ EVs per mL of plasma and/or TNC intensities higher than 200 in IHC measurements of paired tissues). Tissue-EVs were isolated from glioblastoma resections following the protocol from Crescitelli et al.,^66^ with minor modifications. Briefly, the frozen tissue was weighted and transferred to a six-well plate, adding approximately 0.2 g of tissue per well. In each well, 2 mL of RPMI-1640 (Gibco) containing 40 U/mL of DNase I (Sigma) and 2 mg/mL of Collagenase D (Worthington) were added. While on ice, the tissue was gently sliced with a scalpel. The sample was then incubated at 37 °C, for 30 min, while mildly shaking. After 30 min, protease inhibitors (Roche) were added to the solution. The sample was then transferred on top of a 70 μm cell strainer and let pass through gravity. The collected sample was then centrifuged at 300*g* for 10 min at 4 °C and the supernatant was collected and centrifuged at 2,000*g* for 20 min at 4 °C and again 16,500 g for 20 min at 4 °C. The resulting pellet (large EVs, lEVs) was resuspended in 500 μL filtered PBS (with protease inhibitors) and stored at 4 °C, while the supernatant was collected and transferred into a polypropylene centrifuge tube (Beckman Coulter), filled with PBS, and centrifuged at 118,000*g* for 2,5 h at 4 °C. The resulting pellet (small EVs, sEVs) was resuspended in 500 μL filtered PBS (with protease inhibitors). lEVs and sEVs were combined in a single tube and mixed with 3 mL of 60% (wt/vol) OptiPrep (Progen). The 4 mL sample was transferred to the bottom of a new tube and 4 mL of 30% and 4 mL of 10% OptiPrep were layered on top. The gradient was then centrifuged at 186,000*g* for 2,5 h at 4 °C. The purified EVs were collected between the 10% and 30% layer, diluted in PBS and pelleted at 118,000*g* for 2,5 h at 4 °C. The final EVs pellet was resuspended in 100 μL of filtered PBS and immediately used for subsequent analysis.

### Nanosight Tracking Analysis

The concentration and size of EVs was determined by nanoparticle tracking analysis (NTA), using a LM10 instrument (Nanosight, Amesbury, UK). Plasma and CSF EVs were diluted (1:300) in filtered PBS prior to NTA. Five movies of 1 min each were recorded on camera level 15, and then analyzed with detection threshold 4. Plasma- and CSF-EV concentration values were corrected for values per milliliter of sample. For analysis of EVs isolated from tissues, the final EVs resuspension was diluted either 1:1000 or 1:500 in filtered PBS and recorded for 10 times, each video 10 s long, on camera level 15. The analysis was performed by NTA 3.0 software (detection threshold = 6, screen gain = 2). Tissue-EV concentration values were corrected for values per milligram of tissue. All EV size values are presented as mode values.

#### Electron Microscopy

For transmission electron microscopy (TEM) negative stainings, 20 μL of fresh EV samples were adsorbed onto glow-discharged carbon-coated nickel grids (EMS, 400 mesh). The grids were washed three times with PBS, followed by fixation with 2.5% glutaraldehyde (in PBS) for 5 min. Subsequently, the grids were washed five times in deionized water and stained on drops of a solution composed of 1% uranyl acetate and 1% methyl cellulose for 10 min on ice. For immunogold TEM, EV-adsorbed grids were blocked with 1% BSA for 5 min, incubated with the TNC antibody (Novus Bio, clone 4C8MS) for 1 h, washed five times in PBS, incubated with the secondary antibody (Sigma G-7777, goat antimouse, dilution 1:10) for 30 min, and then washed again prior to the glutaraldehyde fixation step. Both immunogold and negatively stained grids were visualized using a FEI Tecnai G20 microscope operated at 80 kV. Images were acquired with a SIS Veleta camera.

For scanning electron microscopy (SEM), grids were processed similarly to the TEM protocol until the fixation step with 2.5% glutaraldehyde. Subsequently, the grids were washed five times in water before air drying and sputtering with 3 nm gold. Gold-coated grids were then analyzed in a Tescan Clara SEM under different conditions to depict specific features more effectively.

### Imaging Flow Cytometry

IFCM was performed to analyze EV tetraspanins (CD9, CD63 and CD81) together with each of the following glioma-related antigens: Tenascin-C (TNC), Integrin beta-1 (ITGB1, also known as CD29), CD44, Profilin-1 (PFN1), Secreted protein acidic and cysteine rich (SPARC), Glycoprotein NMB (GPNMB), CD133 (also known as PROM1), and HLA-DR/DQ/DP (Figure 1A). These surface antigens were selected according to a prior protein screen on glioma cell-derived EVs performed by our group,^9^ and also to their known differential expression in glioblastoma according to TCGA data^34^ (Supporting Information Figure S1).

EVs (3 μL) were stained in 3 μL of filtered PBS containing 8% exosome-depleted FBS (Invitrogen, cat. no. A2720801), with a cocktail of the following antihuman antibodies (3 μL each): PE-conjugated anti-CD9 (Biolegend, clone HI9a, 20 μg/mL - diluted 1:30), FITC-conjugated anti-CD81 (Biolegend, clone 5A6, 200 μg/mL), PacificBlue-conjugated anti-CD63 (Biolegend, clone H5C6, 200 μg/mL), and AlexaFluor647-conjugated (separately) of either anti-TNC (Novus Bio, clone 4C8MS, 0.69 mg/mL), anti-ITGB1 (Biolegend, clone TS2/16, 400 μg/mL), anti-CD44 (Biolegend, clone IM7, 0.5 mg/mL), anti-PFN1 (Novus Bio, #NBP1-19344AF647, 1.06 mg/mL), anti-GPNMB (R&D systems, clone 303,822, 0.2 mg/mL), anti-SPARC (R&D systems, clone 122,511, 0.2 mg/mL), anti-HLA-DR/DQ/DP (Biol + egend, clone 361,704, 100 μg/mL) or anti-CD133 (R&D systems, clone 170,411, 0.2 mg/mL). The 18 μL of EV-cocktail solutions were incubated for 45 min at room temperature (RT) in the dark. Stained EVs were washed with 550 μL of IFCM buffer (filtered PBS containing 2% exosome-depleted FBS) using a 300 kDa filter (Nanosep, 4,000*g* for 7 min at 4 °C), and resuspended in 30 μL of IFCM buffer. IFCM negative controls included cocktails without EVs and with lysed EVs (0.5% of NP40 for 30 min at RT).

Data was acquired on ImageStreamX Mark II Imaging Flow Cytometer (Amnis, Luminex Corporation), for 30 s at 60× magnification, with low flow rate and beads removal. Fluorescent signals were detected for FITC in channel 2 (480–560 nm filter; laser voltage: 150 mW), PE in channel 3 (560–595 nm filter; laser voltage: 100 mW), PacificBlue in channel 7 (435–505 nm filter; laser voltage: 175 mW), and AF647 in channel 11 (642 nm filter; laser voltage: 100 mW). Absolute fluorescence calibration in molecules of equivalent soluble fluorophores (MESF) was performed as previously described.^67^ Nonswarmed results were analyzed using IDEAS software version 6.2 (Amnis, Luminex Corporation) as previously described,^7,8^ and corrected according to their dilutions for values per milliliter of plasma.

### Immunohistochemistry

Immunohistochemistry (IHC) was performed in 30 tumor tissues (from glioblastoma patients of the same EV phenotyping cohort) and 7 non-neoplastic brain tissues as controls (cortex and white matter from temporal lobe epilepsy patients). Tissue samples were fixed in 4% formaldehyde, dehydrated, embedded in paraffin, and sectioned at 2 μm according to standard laboratory protocols. Immunohistochemical staining for Tenascin-C (BC-24, Sigma-Aldrich, 1:1000) was performed on an automated staining machine (Ventana BenchMark TX, Roche Diagnostics, Mannheim, Germany). Detection was performed with diamino-benzidine (DAB) as a chromogen.

Immunostained tissues were digitalized using a Hamamatsu NanoZoomer 2.0-HT C9600 whole slide scanner (Hamamatsu Photonics, Tokyo, Japan). Slide images were exported using NDP view v2.7.43 software. Digital image analysis was performed using ImageJ/Fiji software.^68^ Tissue areas suitable for quantification were labeled via manually drawn regions of interest (ROIs). Tissue areas not eligible for quantification (e.g., due to technical or digital artifacts, large caliber blood vessels) were excluded from the analysis. Normalization was performed by subtraction of the mean value of representative, manually identified background pixels in the grayscale-converted, inverted images. Total tissue areas were measured via consistent global thresholding (0, 233) and subsequent pixel quantification within the ROIs. DAB-positive pixels (i.e., brown immunostaining) were quantified on a three-tiered intensity scale after application of the color deconvolution plugin and background normalization. In detail, pixels were separately quantified within three distinct thresholds [0–100 (strong/3+); 101–170 (medium/2+); and 171–200 (weak/1+)]. Based on the conventional Histo-score, pixel percentages quantities of strong, medium and weak intensity were multiplied by three, two and one, respectively, and then summed up. The generated score (ranging from 0–300) was referred to as a digital histo-score (DH-score).

### dSTORM

EVs isolated from glioblastoma tissues were immunolabeled and imaged using the EV Profiler Kit (#EV-MAN-1.0, ONI) by dSTORM. Briefly, approximately 1 × 10 × 10^12^ EVs were immobilized on microfluidic chips, fixed with F1 solution (provided in the kit) for 10 min, and incubated for 50 min with fluorescently labeled antibodies. The following antibodies were used, either provided in the kit or purchased separately: CD9-CF488 (kit, excitation (ex)/emission (em): 490/515 nm), CD63-CF568 (kit, ex/em: 562/583 nm), CD81-CF647 (kit, ex/em: 650/665 nm), anti-TNC AF647 (ex/em: 650/671 nm, Novus Bio, clone 4C8MS). Finally, samples were again fixed with F1 for 5 min, and a freshly prepared dSTORM-imaging buffer was added prior to image acquisition. Labeled EVs were imaged using the Nanoimager S Mark II microscope (ONI) with 100× oil-immersion objective. Sequential 3-color imaging was performed at 30, 60, and 60% power for the 640, 561, and 488 nm lasers, respectively, at 1000 frames per channel with the angle of illumination set to 53.59°. Prior to the start of the imaging session, channel mapping was calibrated using 0.1 μm TetraSpeck beads (#T7279, Thermo Fisher Scientific). Data were acquired and processed on NimOS software (version 1.19.7, ONI). Subpopulation analyses of EVs that express one, two, or three markers were analyzed using ONI’s online platform CODI (https://alto.codi.bio). Herein, a density-based clustering analysis with drift correction and filtering was performed to evaluate the biomarker positivity at a single-particle level.

### Single Cell RNA-Sequencing Analysis

For single cell analysis we used GBMap^37^ reference data set. Single cell data were processed using the Seurat (v5.0) package and r software.

### Spatial Transcriptomics Analysis

For spatial data analysis, we acquired the spatially resolved RNA-seq data sets using the SPATAData package (https://github.com/theMILOlab/SPATAData).^38,69^ Data processing and visualization were performed by the SPATA2 package (https://github.com/theMILOlab/SPATA2). Spatial multiomics data was originally acquired for Ravi et al.^38^ and adapted for this study. We implemented the copy number alterations by the runCNV() function of SPATA2.

### Spatial Trajectory Analysis

We performed spatial trajectory analysis by the createSpatialTrajectories function from the SPATA2 package. We selected tumor tissue based on the chromosomal gain of 7 and loss of 10 as a StartPoint and normal neuronal tissue (no CNA) as the end point. Four different samples out of the Freiburg spatial cohort were selected and horizontally integrated by the distance. Gene expression heatmaps were ordered by the maximal expression based on the plotTrajectoryHeatmap() function. Spatial changes of chromosomal alterations (7 and 10) were added to the plot by the plotTrajectoryLineplot() function.

### Genome Wide Methylation Array and Patient’s Classification

Genomic DNA was isolated from glioblastoma tissue using the NucleoSpin tissue kit (Macherey Nagel) and followed to methylation analysis with the Infinium Human Methylation 850 K arrays (Illumina Inc., USA). Bisulfite treatment, whole-genome DNA amplification, hybridization and single-base extension, fluorescence staining, and scanning of the chips were performed following the manufacturer’s instructions. Genome-wide DNA methylation profiles were analyzed as previously described,^9,70^ to obtain epigenetic tumor classifications (RTK-I, RTK-II and MES) using the DKFZ brain tumor classifier (versions V11b4 and V12.8, available in https://www.molecularneuropathology.org/mnp).

### Enrichment of TNC Positive EVs

Glioblastoma patients tested as positive for the TERT*C228T promoter mutation were selected for this analysis. EVs were purified from 12 mL (single samples) or up to 30 mL (pooled samples) of plasma by dUC and resuspended in filtered PBS containing protease/phosphatase inhibitors. In addition to plasma samples, EVs derived from glioblastoma-tissue resections (unmatched) were also included in analysis. Half of EVs were directly proceeded to DNA isolation (for total EV DNA), and the other half to magnetic sorting (MACS) for enrichment of TNC^±^ fractions. EVs were maintained on ice during the whole experiment. All incubations and centrifugations were performed at 4 °C.

For the TNC magnetic sorting, EVs were prior stained overnight in a solution containing 1 μL of human Fc blocking reagent (Miltenyi Biotec, cat no. 130-059-901), 3 μL of 8% FCS and 7 μL of anti-TNC antibody (Novus Bio, clone 4C8MS, undiluted) conjugated with AF647 fluorophore. Next, the stained EVs were washed with 550 μL of 2% FCS, using a 300 kDa filter (Nanosep, 4,000*g* for 60–120 min). A second washing step was repeated using 300 μL of MACS buffer (filtered PBS containing 0.5% bovine serum albumin and 2 mM EDTA). The washed EVs were resuspended from the filter with 50 μL of MACS buffer by carefully pipetting up and down, and then incubated with 50 μL of anti-AF647 magnetic beads (Anti-Cy5/Anti-Alexa Fluor 647 MicroBeads, Miltenyi Biotec, cat no. 130-091-395) for 1 h. The samples proceed to magnetic separation through a MACS MS magnetic column (Miltenyi Biotec), in order to separate the TNC^+^ EVs (on magnetic beads) from the negative fraction (flow-through). The binding success between EVs and magnetic beads were visualized by SEM. TNC enrichment in the positive sorted fraction was confirmed both by conventional FACS (CytoFLEX, Amnis) and IFCM. The enriched fractions for TNC^+^ and TNC^–^ EVs were followed to DNA isolation.

### Droplet Digital PCR

DNA was extracted from total and sorted EVs (TNC^+^ and TNC^–^ fractions) using the MasterPure total DNA and RNA isolation kit (Biosearch Technologies) according to the manufacturer’s instructions for total RNA removal, and eluted in 10 μL of DNase-free water. For comparison purposes, cfDNA was obtained from 0.6 to 1.2 mL of total plasma using the MagMax cfDNA Isolation Kit (Applied Biosystems).

The purified DNA (undetectable up to 8 ng) was used in droplet digital PCR (ddPCR), in order to detect *TERT C228T* mutation (Bio-Rad, dHsaEXD72405942 assay, cat no. 12003908), according to the manufacturer’s protocol. Briefly, 5 μL of DNA were mixed with 1.5 μL of water, 10 μL of ddPCR Supermix for probes (no dUTP), 1 μL of the assay probes (containing primers for *TERT* promoter copies of *C228T* [FAM probes] and *C228* WT [HEX probes]), 2 μL of 5 M Betaine solution (Sigma-Aldrich, cat. B0300), 0.25 μL of EDTA (0.5M, pH 8.0; ThermoFisher Scientific, cat. AM9260G), and 0.25 μL of CviQi restriction enzyme. The 20 μL of ddPCR reaction mix were then followed to generation of droplets, according to the standard ddPCR procedures using the QX200 ddPCR system (Bio-Rad), and an adapted PCR cycling consisting of a 5 min step at 95 °C, followed by 50 cycles of 96 °C for 30 s and hybridization temperatures (gradient: 12 cycles of 62 °C and 40 cycles of 58 °C) for 1 min; terminating with a cycle of 10 min at 98 °C. Droplets containing the amplicons were counted by a QX200 Droplet reader and analyzed by QuantaSoft analysis software. Negative, positive, and nontemplate controls were included in all experiments. Samples with less than 30 positive droplets for the wild-type amplicons were excluded from analysis. In the end, results from 21 TNC^+^ EVs, 19 TNC^–^ EVs, 21 total EVs and 31 cfDNA samples from plasma, as well as 9 TNC^+^ and TNC^–^ paired EVs from glioblastoma tissues, proceeded to statistical analysis.

### Statistical Analysis

IFCM acquired data was analyzed using the IDEAS software version 6.2 (Amnis, Luminex Corporation), as previously described.^7^ EV counts/mL were obtained for each protein combination and compared among the groups by nonparametric *t* tests (Mann–Whitney for unpaired comparisons, Wilcoxon for paired and Kruskal–Wallis with FDR correction for multiple comparisons). Outliers were identified by the ROUT method (with *Q* = 0.1%, for identification of definitive outliers), and when applicable, removed prior *t* tests analyses. Spearman correlation and linear regressions (in normal or categorized values) were made to compare the values with clinical parameters. All analyses were performed using GraphPad Prism Software (v. 9.1.2). For ddPCR analyses, the fraction of mutated and wild-type positive droplets were used to estimate the frequency of TERT*C228T mutation according to Poisson distribution, with the QuantaSoft analysis software (Bio-Rad). The fractional abundances were analyzed by nonparametric *t* tests, in order to compare the detected TERT*C228T frequencies in DNA of TNC^+^, TNC^–^ and total EVs, as well as in bulk cfDNA.

## Supporting Information Available

Supporting Information Supporting Information The Supporting Information is available free of charge at https://pubs.acs.org/doi/10.1021/acsnano.4c13599.

Tables S1–S3: clinical and demographic description of patient’s cohorts, statistical data of plasma-EVs, statistical data of tissue-EVs (XLSX)
Figures S1–S6: expression and overall survival of evaluated markers, EV expression of TNC/CD81 and TNC/CD63 populations, TNC levels in cells and EVs from matched tissues, levels of tissue-EV populations by ONI analysis, spatial transcriptomics, and characterization of TNC magnetic sortings (PDF)
