# Supplementary material for: Extracellular Vesicles Carrying Tenascin-C are Clinical Biomarkers and Improve Tumor-Derived DNA Analysis in Glioblastoma Patients
Source: ACS Nano. 2025 Mar 8;19(10):9844–59. doi: 10.1021/acsnano.4c13599 (PMC11924321; doi:10.1021/acsnano.4c13599)
Supplement: Supplementary file 2 — nn4c13599_si_002.pdf [file nn4c13599_si_002.pdf]

SUPPORTING INFORMATION FOR PUBLICATION

Extracellular vesicles carrying Tenascin-C are clinical biomarkers and improve tumor-derived DNA analysis in glioblastoma patients

Amanda Salviano-Silva[1*], Kathrin Wollmann[1], Santra Brenna[2], Rudolph Reimer[3], Julia E. Neumann[4,5], Matthias Dottermusch[4,5], Laura Woythe[6], Cecile L. Maire[1], Berta Puig[2], Ulrich Schüller[4,7,8], Meike J. Saul[9], Manfred Westphal[1], Richard Drexler[1], Lasse Dührsen[1], Jens Gempt[1], Dieter H. Heiland[10,11,12,13,14], Katrin Lamszus[1], Franz L. Ricklefs[1*†]

[1] Department of Neurosurgery, University Medical Center Hamburg-Eppendorf, Hamburg, 20246, Germany
[2] Neurology Department, Experimental Research in Stroke and Inflammation, University Medical Center Hamburg-Eppendorf, Hamburg, 20246, Germany
[3] Leibniz Institute for Experimental Virology, Hamburg, 20251, Germany
[4] Institute of Neuropathology, University Medical Center Hamburg-Eppendorf, Hamburg, 20246, Germany
[5] Center for Molecular Neurobiology (ZMNH), University Medical Center Hamburg-Eppendorf, Hamburg, 20246, Germany
[6] Oxford Nanoimaging Limited (ONI), Oxford OX2 8TA, United Kingdom
[7] Department of Pediatric Hematology and Oncology, University Medical Center Hamburg-Eppendorf, 20246, Hamburg, Germany
[8] Children's Cancer Research Center Hamburg, Hamburg, 20246, Germany
[9] Department of Oncology, Hematology and Bone Marrow Transplantation with Section Pneumology, University Cancer Center Hamburg, University Clinic Hamburg-Eppendorf, Hamburg, 20246, Germany.
[10] Department of Neurosurgery, Medical Center University of Freiburg, Freiburg, D-79106, Germany
[11] Translational Neurosurgery, Friedrich-Alexander University Erlangen Nuremberg, Erlangen, 91054, Germany
[12] Department of Neurosurgery, University Hospital Erlangen, Friedrich-Alexander University Erlangen Nuremberg, Erlangen, 91054, Germany
[13] Department of Neurological Surgery, Northwestern University Feinberg School of Medicine, Chicago, IL, 60611, USA
[14] German Cancer Consortium (DKTK), partner site Freiburg, Freiburg, D-79106, Germany.
† Senior author

*** Corresponding authors:** Franz Ricklefs and Amanda Salviano-Silva. Department of Neurosurgery, University Medical Center Hamburg-Eppendorf, Martinistrasse 52, 20246 Hamburg, Germany. E-mail: f.ricklefs@uke.de and a.salvianodasilva@uke.de

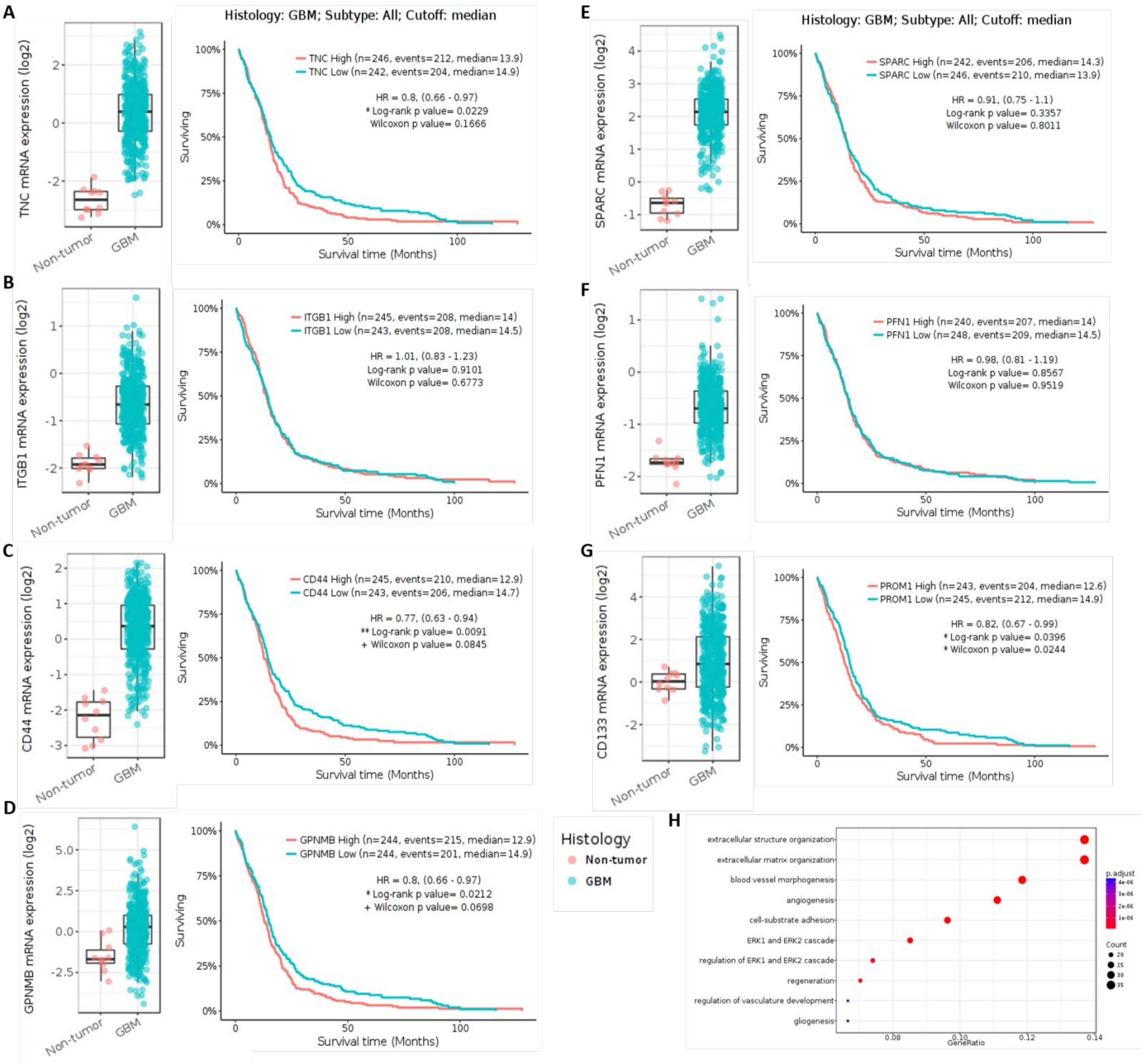

Supp. Figure 1. Differential expression levels and overall survival for the selected candidate markers in glioblastoma tissue, according to TCGA data.

(A-G) Differential expression levels and overall survival graphs for the antigens selected to be investigated in glioblastoma EVs in this study: TNC, ITGB1, CD44, GPNMB, SPARC, PFN1 and CD133. No data was available for HLA-DR, HLA-DQ or HLA-DP. Data obtained from TCGA (platform HG-U133A), using the Gliovis Portal.[34]

(H) Gene ontology analysis of TNC biological process in glioblastoma. Data obtained from TCGA (platform HG-U133A).[34]

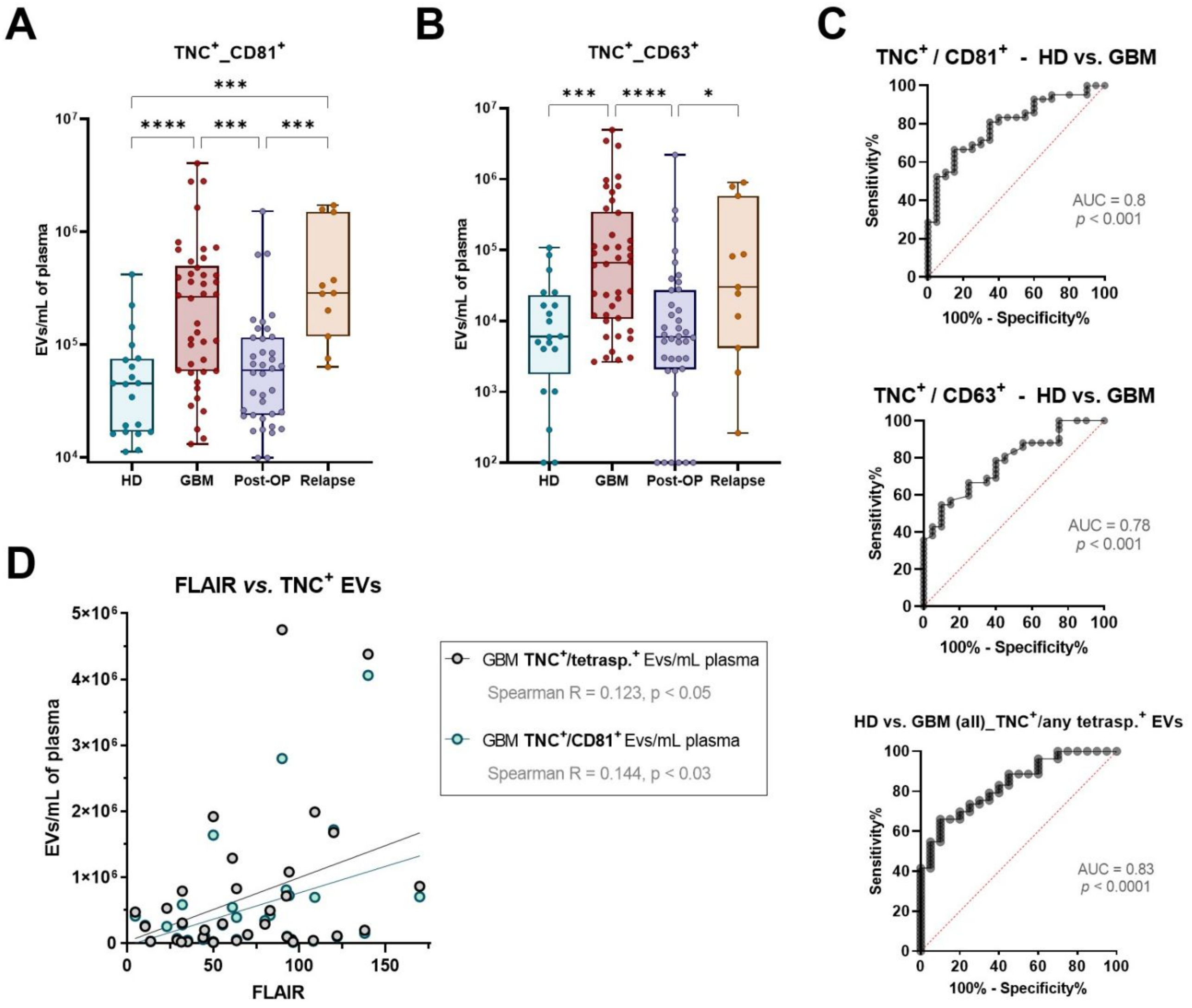

Supp. Figure 2. Differential levels of TNC$^+$/CD81$^+$ and TNC$^+$/CD63$^+$ plasma EVs in glioblastoma.

(A) TNC$^+$/CD81$^+$ EVs are elevated in plasma of newly diagnosed (FC = 5.9) and recurrent (FC = 6.4) glioblastoma patients, in comparison to HD subjects. TNC$^+$/CD81$^+$ EVs are also 4.5- and 4.9-fold decreased in post-OP individuals, when respectively compared with newly diagnosed and recurrent patients.

(B) TNC$^+$/CD63$^+$ EVs are elevated in plasma of newly diagnosed patients (FC = 10.96), in comparison to HD subjects. TNC$^+$/CD63$^+$ EVs are also 11.06- and 5.06-fold decreased in post-OP individuals, when respectively compared with newly diagnosed and recurrent patients.

(C) ROC graphs with statistically significant AUC values for: TNC$^+$/CD81$^+$ and TNC$^+$/CD63$^+$ EVs for HD and newly diagnosed glioblastoma patients (upper and middle panels); and for TNC$^+$ EVs (regardless the tetraspanin analyzed) between HD and glioblastoma patients (newly diagnosed and recurrent altogether) (panel below).

(D) TNC$^+$/CD81$^+$ EVs per mL of plasma correlate with peritumoral fluid-attenuated inversion recovery (FLAIR) hyperintensity, obtained from MRI scans.

* = p < .05, ** = p < .01, *** = p < .001, **** = p < .0001.

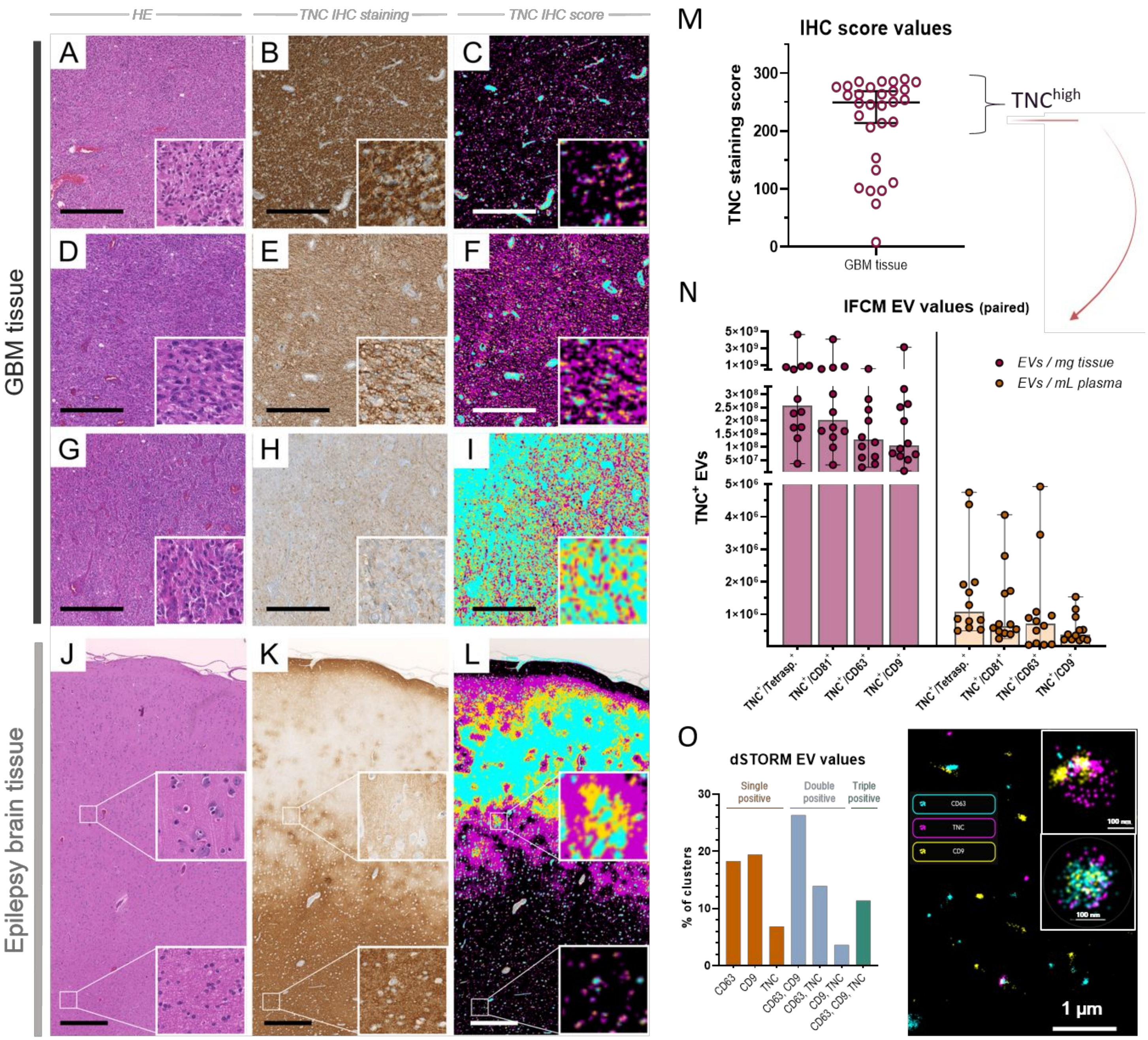

Supp. Figure 3. TNC protein expression in glioblastoma and non-tumor brain tissue.

(A-L) TNC-immunostaining in glioblastoma tissues (n = 30; paired with plasma samples) and non-neoplastic brain tissue from epilepsy control subjects (n = 7). Histological images of GBM (A – I) and non-neoplastic brain tissue (J - L) stained for H&E (left column) and TNC expression via immunohistochemistry (middle and right column). The right column shows the color-coded immunostaining pixel intensities, which provided the basis for immunostaining quantification (turquoise = no signal; yellow = weak signal; pink = moderate signal, black = strong signal), used for calculating the digital histo-score (DH-score, ranging from 0 – 300). Representative images are shown of a glioblastoma with high (A-C), medium (D – F), and low (G – I) immunostaining intensities. Non-neoplastic brain tissue exhibits mostly weak to moderate staining signals in the cortex (J – L, upper insets) and mostly strong staining signal in the white matter (J-L, lower insets). Note the common perinuclear clearing and accentuation of the cell periphery in tumor cells (insets A – I) and non-neoplastic glial cells and neurons (insets J – L). Scale bar is 500 µm in all images.

(M) TNC immunostaining score values of all analyzed glioblastoma tissues. Most of evaluated samples had DH-scores greater than 200 (major color-coded in black, as exemplified in A-C). Only one tissue presented very low TNC staining signals (DH-score = 8) (shown in G-I). All DH-scores are described in Supp. Table 3.

(N) TNC^+ EV subpopulations from paired samples presenting high TNC score in IHC (DH-score > 200, as represented in figure A-C). On the left side of the graph (purple dots), are represented the levels per milligram of tissue of TNC^+ EVs isolated from glioblastoma tissues (n = 12). On the right side (orange dots) are shown their paired levels per milligram of plasma. All TNC^+ EV subpopulation values from tumor tissues are described in Supp. Table 3.

(O) dSTORM characterization with a sample of glioblastoma tissue-EVs. The percentage of analyzed clusters containing single-, double- and triple positive EVs for CD63, CD9 and TNC are represented in the graph. On the right, cluster overview (1 µm) and close-up images (100 nm) are shown for tissue-EVs expressing CD63 (cyan), CD9 (yellow) and TNC (magenta). The TNC, CD9 and CD63 EV subpopulation values from a tumor tissue sample are described in Supp. Figure 4, in addition to CD81, CD9 and CD63 EV values of a second tissue sample.

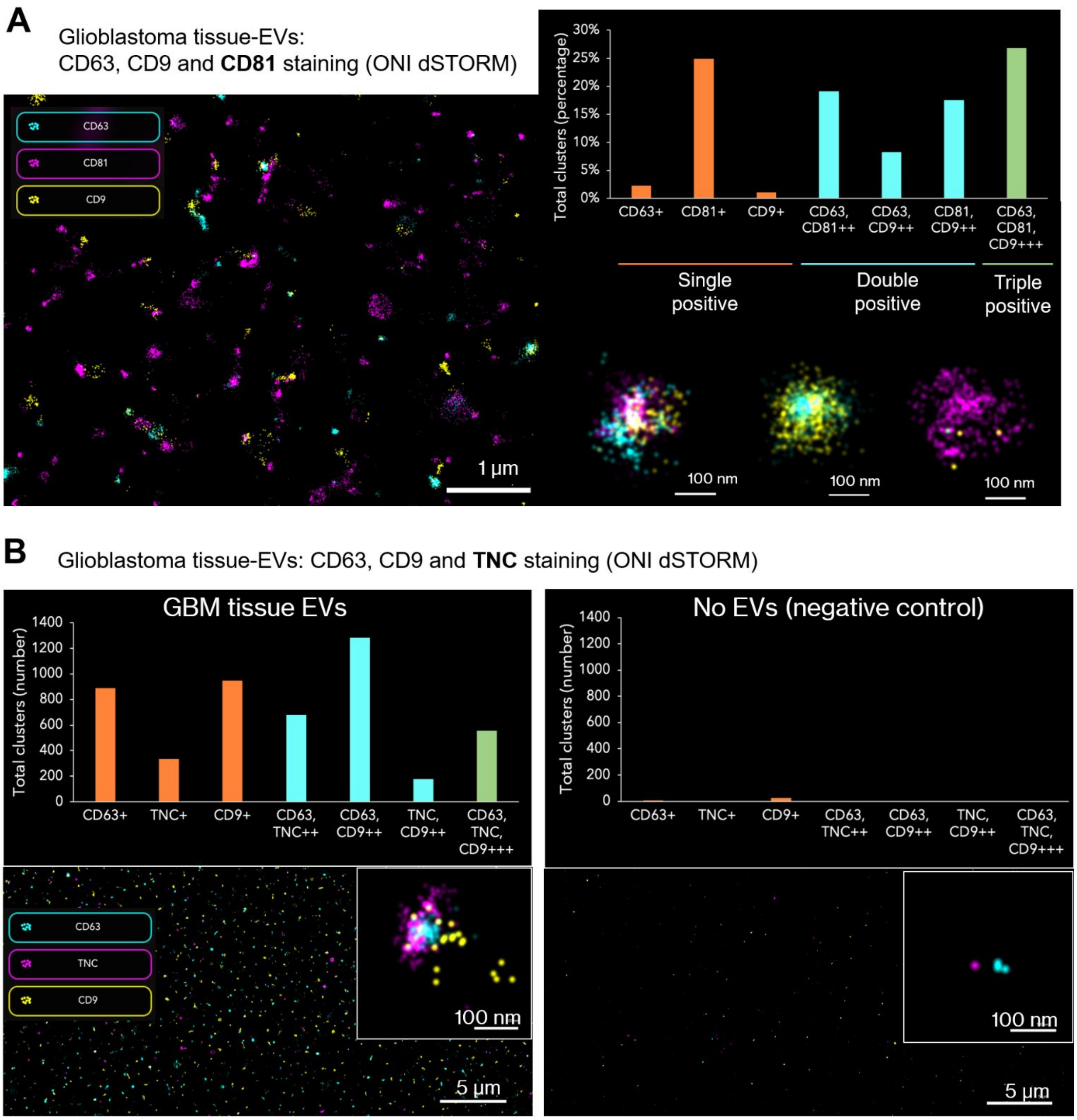

Supp. Figure 4. dSTORM characterization of tetraspanins and TNC in glioblastoma tissue-derived EVs.

(A) Cluster overview (1 µm) and close-up images (100 nm) of EVs expressing CD63 (cyan), CD9 (yellow) and CD81 (magenta) tetraspanins, in a sample of tissue-derived EVs. The percentage of analyzed clusters containing single-, double- and triple positive EVs for CD63, CD9 and CD81 are represented in the graph (right-up).

(B) Left-up: absolute counts of total analyzed clusters of single-, double- and triple positive EVs for CD63, CD9 and TNC. Left-down: cluster overview (5 µm) and close-up images (100 nm) of EVs expressing CD63 (cyan), CD9 (yellow) and TNC (magenta), in a sample of tissue-derived EVs. Right: negative control (experiment performed without EVs), showing no substantial cluster counts (up) and cleaner background overview (down).

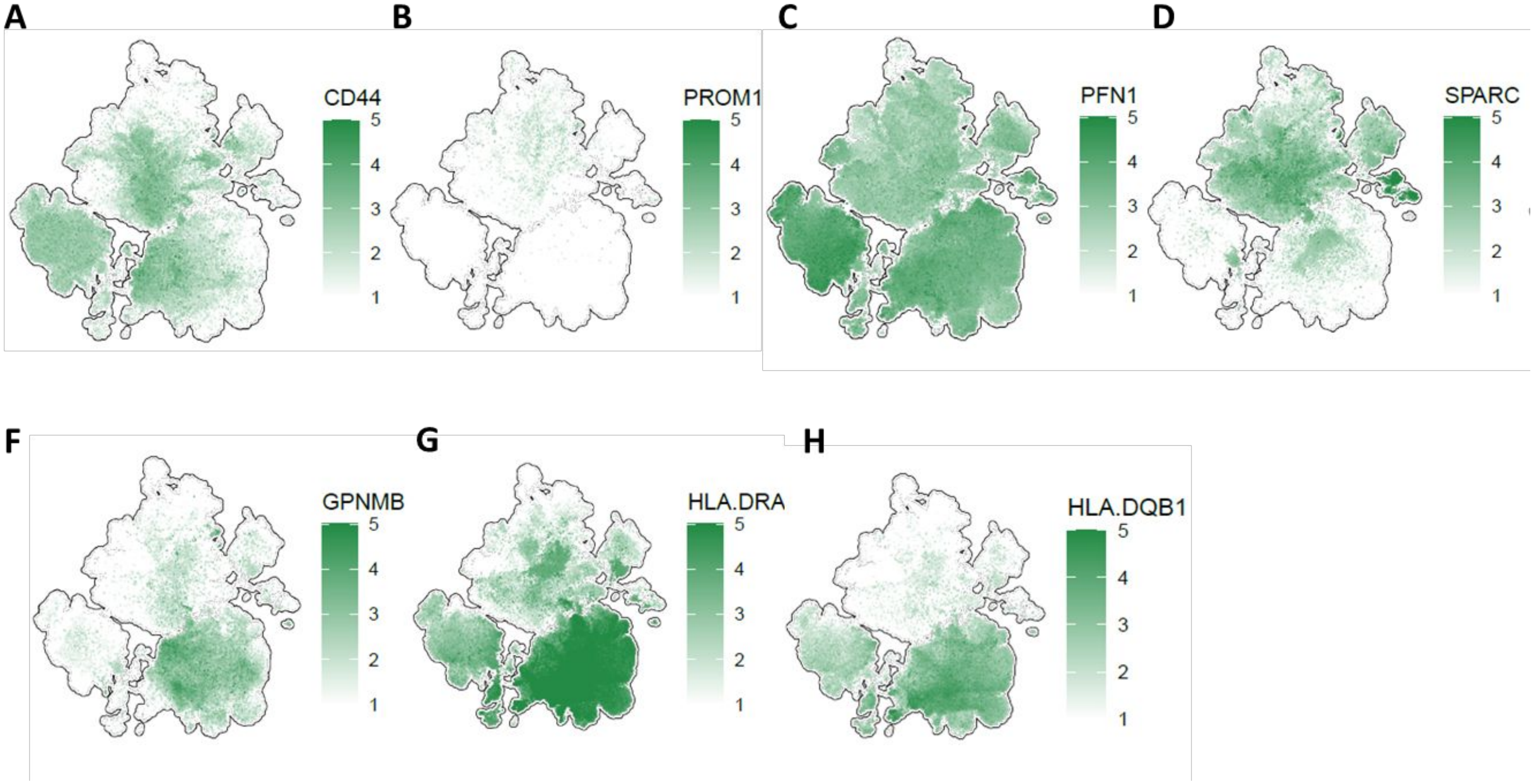

Supp. Figure 5. RNA levels of investigated glioblastoma-associated markers in tumor tissue, by spatial transcriptomics.

RNA levels from all markers investigated in the present study, in different regions of the tumor tissue. The tissue subpopulations are illustrated in the figure 5 A of the manuscript.

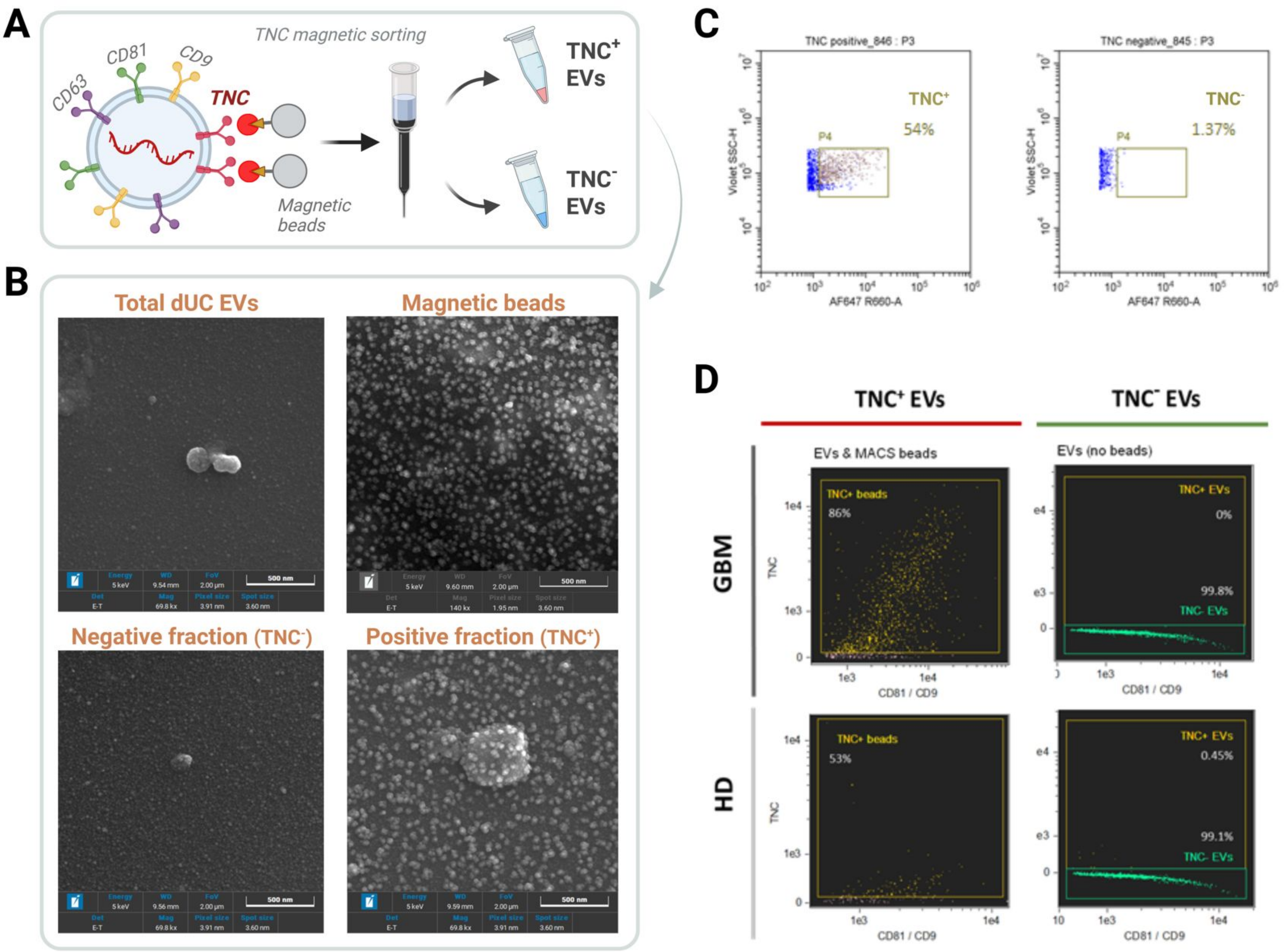

Supp. Figure 6. Workflow and evaluation of TNC enrichment in MACS sorted EVs from plasma.

(A) TNC enrichment workflow, where differential ultracentrifuged EVs (dUC EVs) are submitted to indirect magnetic sortings for obtention of TNC^+ and TNC^- EV fractions.

(B) Scanning electron microscopy of total dUC EVs (upper left), magnetic beads used for enrichment (upper right) and the different EV fractions obtained after TNC magnetic sorting (below).

(C) Flow cytometry (CytoFLEX) analysis of TNC^+ and TNC^- EVs, showing an enrichment of TNC in the positively sorted fraction (54% in TNC^+), in comparison to the negative fraction (1.37% in TNC^-).

(D) Imaging flow cytometry (ImageStream) analysis of TNC^+ and TNC^- EVs from a glioblastoma patient and a HD subject, additionally proving the enrichment of TNC in the positively sorted fraction (86% and 53%, respectively), in comparison to the negative fraction (0% and 0.45%, respectively).
